# Biallelic *GLTP* mutations cause nonsyndromic epidermal differentiation disorder via disrupted epidermal glucosylceramide transport

**DOI:** 10.1172/JCI198835

**Published:** 2026-02-05

**Authors:** Zeqiao Zhang, Shimiao Huang, Adam Jackson, Elizabeth A Jones, Siddharth Banka, Chao Yang, Sisi Zhao, Kunlun Lv, Sha Peng, Zhimiao Lin, Huijun Wang

**Affiliations:** 1 Dermatology Hospital, and; 2 The First School of Clinical Medicine, Southern Medical University, Guangzhou, China.; 3 Division of Evolution, Infection and Genomics, School of Biological Sciences, Faculty of Biology, Medicine and Health, University of Manchester, Manchester, United Kingdom.; 4 Manchester Centre for Genomic Medicine, St. Mary’s Hospital, Manchester University NHS Foundation Trust, Health Innovation Manchester, Manchester, United Kingdom.

**Keywords:** Dermatology, Genetics, Metabolism, Autophagy, Lipidomics, Monogenic diseases

## Abstract

Ceramides are essential skin lipids for maintaining the mammalian skin permeability barrier, which protects against external stimuli. The precursor of epidermal ceramides, glucosylceramides (GlcCer), is synthesized within granular keratinocytes while its precise cellular transport mechanisms remain poorly characterized. Here, we identified 3 pathogenic variants in the *GLTP* gene, which encodes glycolipid transfer protein, in 5 unrelated families with nonsyndromic epidermal differentiation disorder presenting with generalized skin scaling. The biallelic *GLTP* variants resulted in loss of competent GLTP expression. CRISPR/Cas9-generated *Gltp-*knockout mice exhibited lethal barrier defects, partially recapitulating the clinical features of our patients. We demonstrated that GLTP facilitated GlcCer transport in differentiated keratinocytes, with its deficiency causing impaired GlcCer trafficking and consequent aberrant retention in lysosomes, thereby disrupting lysosome function. The lysosomal dysfunction impaired autophagy flux, resulting in delayed keratinocyte terminal differentiation, which is expected to compromise the skin barrier integrity and ultimately lead to abnormal scaling. Pharmacological inhibition of GlcCer synthesis effectively rescued both autophagy and keratinocyte differentiation defects. Our findings establish *GLTP* as a novel underlying gene for nonsyndromic epidermal differentiation disorders and unravel its essential role in maintaining skin homeostasis during terminal differentiation by mediating epidermal GlcCer transport.

## Abstract 2

<strong>GLTP gene defects disrupt skin lipid transport, causing a severe scaling disorder (ichthyosis), revealing a key mechanism for skin barrier formation and potential treatment targets.</strong>

## Introduction

The integumentary system comprising the skin and its appendages forms the essential interface between organisms and the environment. To prevent dehydration and repel threats, terrestrial organisms evolve an effective epidermal barrier. In humans, this barrier serves as the first line of body defense, maintaining skin homeostasis by minimizing water loss and protecting against microbial and other environmental insults (1). This intricate system originates from the basal keratinocytes, which progressively differentiate as they migrate upward (2). Upon reaching the stratum corneum (SC), these cells transform into enucleated corneocytes, each encapsulated within a robust, loricrin-rich cornified envelope. Externally, this envelope is bound to a specialized corneocyte lipid envelope and embedded in a dense, lamellar intercellular lipid matrix (3), an architecture that confers the barrier cohesion and impermeability. The SC lipid matrix is predominantly composed of ceramides, along with cholesterol and free fatty acids (4, 5). Ceramide biosynthesis initiates in the endoplasmic reticulum (ER), followed by trafficking to the Golgi apparatus for conversion into glucosylceramides (GlcCer) and sphingomyelins (5). Unlike other cell types, where GlcCer is processed into complex glycosphingolipids in the Golgi apparatus or hydrolyzed in lysosomes (6), epidermal GlcCer is transported to the stratum granulosum-SC (SG-SC) interface for hydrolysis into ceramide, a specialized pathway essential for SC barrier function. Current studies have established that GlcCer is stored in the lamellar bodies (LBs) of the granular keratinocytes and is subsequently transported extracellularly through LB extrusion (4, 7, 8), yet, the specific molecular mechanisms orchestrating this transport are not fully elucidated.

Previous studies have identified 3 intracellular transporters for GlcCer, including 4-phosphate adaptor protein 2 (FAPP2), ATP-binding cassette sub-family A member 12 (ABCA12), and glycolipid transfer protein (GLTP) (8–10). GLTP is a cytosolic protein known for its role as both a sensor of glycolipid and a lipid transporter (11). While in vitro studies have implicated GLTP’s ability in mediating the transfer of various glycosphingolipids including GlcCer, galactosylceramide, lactosylceramide, and monosialoganglioside between artificial lipid membranes (9, 12, 13), its physiological functions remain incompletely understood. Recently, evidence from mouse models indicated that GLTP could mediate galactosylceramide transfer from the ER to myelin membranes in oligodendrocytes, thereby regulating myelin outgrowth and oligodendrocyte maturation (14, 15). Notably, despite its high expression in skin and correlative associations with epidermal differentiation and skin barrier repair (16), the precise physical function of GLTP in epidermis has not been fully elucidated.

Congenital defects in the skin barrier may lead to a group of epidermal differentiation disorders, previously known as congenital ichthyosis (17, 18). Mutations in at least 13 genes are responsible for autosomal recessive congenital ichthyosis, with the majority of these genes involved in lipid synthesis, processing, or transport pathways. Nevertheless, the genetic basis remains unresolved in approximately 15% of affected individuals (19). In the present study, we identified 3 germline *GLTP* variants in 5 unrelated families with nonsyndromic epidermal differentiation disorder (nEDD) (20). The 6 affected individuals presented with generalized scaling of skin with or without erythema. We showed that GLTP is necessary for efficient GlcCer trafficking in differentiated keratinocytes. Defective GLTP expression resulted in impaired GlcCer trafficking and consequent aberrant lipid retention in the lysosome, which impaired autophagy flux, delayed terminal differentiation of the keratinocytes, and compromised skin barrier integrity, ultimately leading to abnormal scaling.

## Results

### Identification of GLTP variants in 6 nEDD patients.

**Figure 1:**
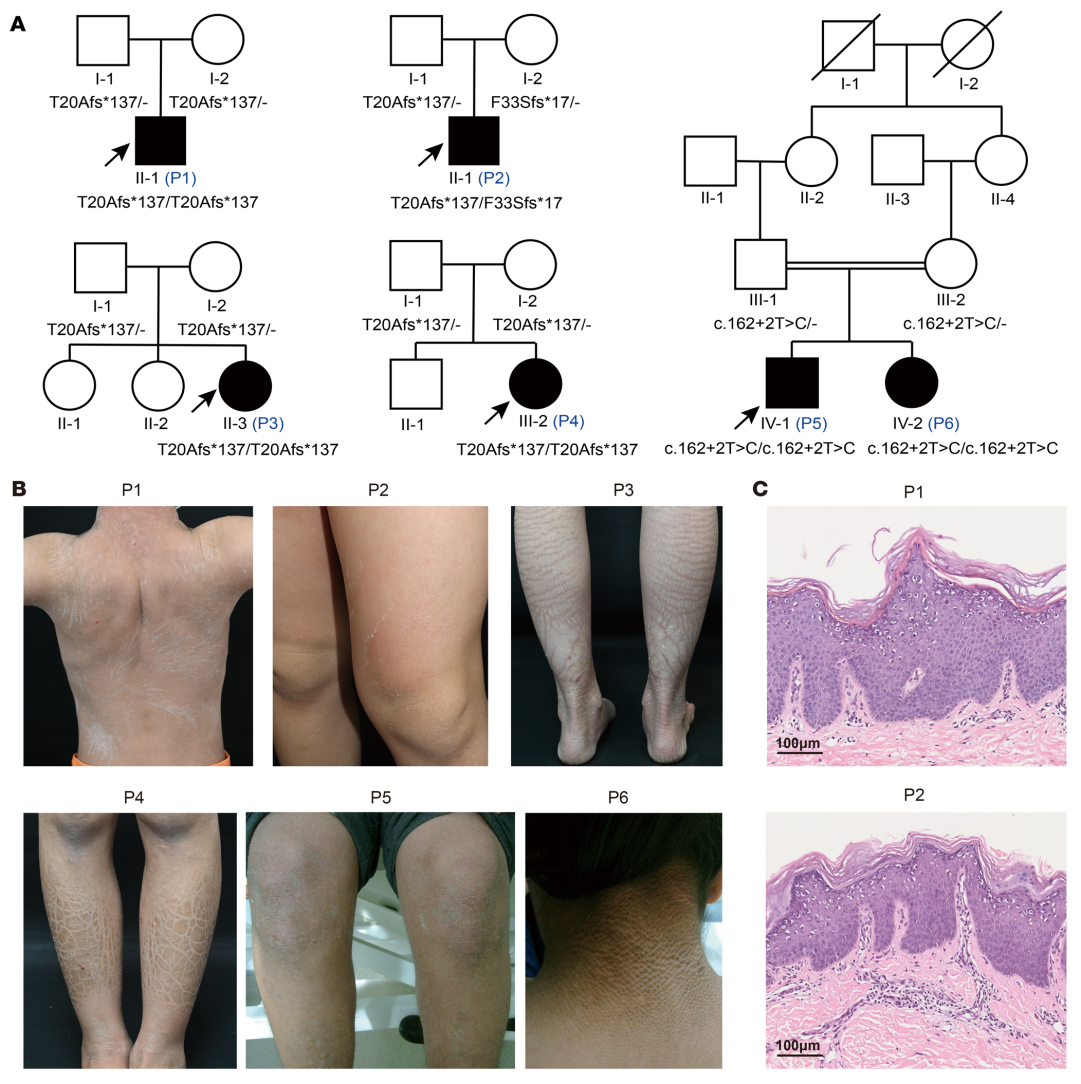
Clinical manifestations and histopathological findings of patients with nEDD. (**A**) Pedigrees of the 5 nEDD families with individuals (solid symbols) carrying biallelic *GLTP* variants. Slashes, deceased individuals; double horizontal lines, consanguinity; arrows, probands. (**B**) P1 exhibits generalized, brownish-to-gray scales on the neck and trunk. P2 shows dry, thickened skin with fine scales on the legs. Large, plate-like, geometric scales are prominent on the legs of P3 and P4. Affected family members (P5, P6) demonstrate widespread ichthyosis featuring marked hyperkeratosis and hyperpigmentation in flexural areas. (**C**) H&E staining of skin biopsies from P1 and P2 shows hyperplasia with focal parakeratosis, thickened stratum granulosum layer with numerous intracellular vacuoles, and mild perivascular lymphocytic infiltration in the superficial dermis. Scale bars: 100 μm.

We investigated four unrelated individuals with nEDD (P1–P4) of Chinese Han ancestry (Figure 1A). All affected individuals displayed generalized dry, scaly skin and erythema at birth or soon after. Their condition progressively evolved into diffuse hyperkeratosis and desquamation, predominantly affecting body folds and extensor surfaces of the extremities, accompanied by mild to severe pruritus (Figure 1B). The symptoms demonstrated seasonal variation, with exacerbation during winter and amelioration in warmer weather. Notably, individual P2 exhibited a relatively milder phenotype than the other affected individuals. None of the affected individuals exhibited collodion membrane at birth or demonstrated abnormalities involving teeth, hair, or other systems. Histological examination of lesional skin of P1 and P2 showed marked compact hyperkeratosis with focal parakeratosis, acanthosis, thickening of the granular layer, and mild perivascular lymphocytic infiltration in the superficial dermis (Figure 1C). Notably, numerous cytoplasmic vacuoles in the granular layer were observed.

**Figure 2:**
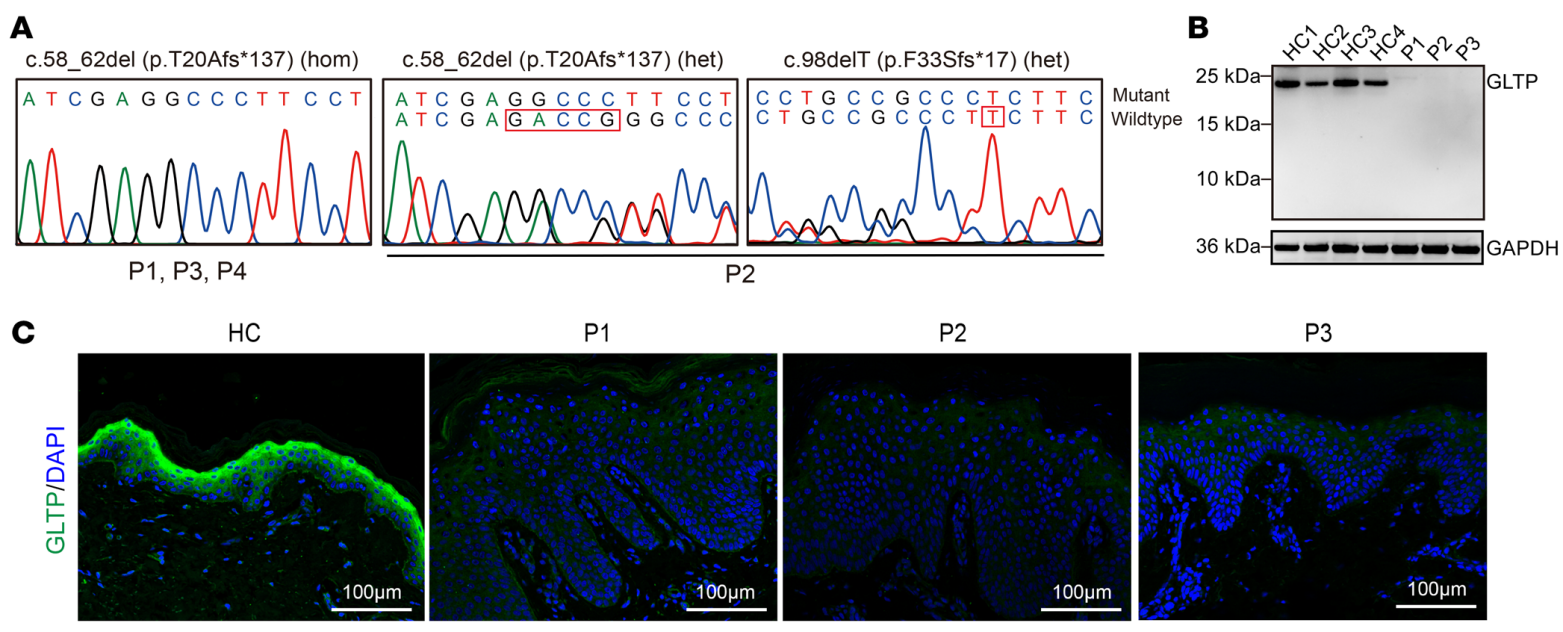
Genetic sequencing and protein expression analysis of GLTP. (**A**) Sequence chromatograms show homozygous or compound heterozygous *GLTP* variants in patients. Deleted bases are marked in red frames. (**B**) Western blot analysis demonstrates absent GLTP expression in patient-derived keratinocytes (P1–P3) compared with 4 healthy controls (HC). (**C**) Representative immunofluorescence image shows absent or weak GLTP signal in the epidermis of lesional skin of the patients (P1, P2, and P3), contrasting with the pronounced staining in the suprabasal epidermis of the HC skin. Scale bars: 100 μm.

To identify candidate pathogenic variants underlying their presentation, we performed trio whole-exome sequencing in 3 unrelated families indexed by their respective probands (P1–P3) and focused on genes with rare (minor allele frequency, MAF < 0.005) variants, including homozygous, compound heterozygous, or de novo variants present in at least 2 patients. Only one gene, *GLTP,* fulfilled these criteria. Both individuals P1 and P3 were homozygous for a frameshift variant (NM_016433.4: c.58_62del, p.T20Afs*137) in exon 1 of the *GLTP* gene (Figure 2A). Compound heterozygous frameshift variants (NM_016433.4: c.58_62del, p.T20Afs*137 and c.98delT, p.F33Sfs*17) in exon 1 of *GLTP* were identified in P2 (Figure 2A). Sanger sequencing confirmed cosegregation of the variants with the phenotype in all these families (Figure 2A). Notably, P3 harbored an additional heterozygous pathogenic variant c.3321del (p.G1109Efs*13) in the *FLG* gene (NM_002016.2), a known causative gene for ichthyosis vulgaris that is likely to exacerbate the phenotype. Otherwise, no disease-causing variants in known causative genes of ichthyosis were detected in the patients. The c.98delT variant is not recorded in any public databases, while the c.58_62del variant is recorded at a frequency of 0.0002932 (13 out of 44,336; rs778854058) in the East Asian population (https://gnomad.broadinstitute.org). Additionally, Sanger sequencing for the entire *GLTP* coding region in P4 identified the same homozygous c.58_62del variant.

Via GeneMatcher (21), we identified another sibling pair (P5 and P6) with a homozygous *GLTP* splice site variant c.162+2T>C. This variant was identified through an independent reanalysis of genome sequencing data from the 100,000 Genomes Project (22), utilizing a previously described family-based, panel-agnostic bioinformatics pipeline (23–25). The siblings were born to consanguineous parents of Pakistani ancestry and had a clinical diagnosis of nEDD with more pronounced skin hyperkeratosis around the neck and the cubital fossa compared with P1–P4 (Figure 1B). The c.162+2T>C variant was recorded at a frequency of 0.00002197 (2 out of 91034; rs751772277) in the South Asian population (https://gnomad.broadinstitute.org). Both parents are heterozygous for the variant. RT-PCR of peripheral blood-derived RNA from P5 and the heterozygous parent revealed skipping of exon 2, which leads to a frameshift and premature termination of the *GLTP* transcript (p.D35Efs*103) (Supplemental Figure 1A; supplemental material available online with this article; https://doi.org/10.1172/JCI198835DS1).

Collectively, these results indicate that bi-allelic *GLTP* loss-of-function variants are potentially a novel cause of nEDD.

### Loss of competent GLTP expression resulted from the truncating GLTP variants.

In humans, GLTP is almost ubiquitously expressed, with the highest expression in skin, brain, and esophagus (Human Protein Atlas; https://www.proteinatlas.org/ENSG00000139433-GLTP/tissue). To investigate the impact of the frameshift variants on *GLTP* mRNA expression, we performed reverse transcription quantitative real-time PCR (RT-qPCR) on mRNA extracted from the primary keratinocytes of P1, P2, and P3. The results showed that the *GLTP* mRNA level was not significantly reduced in the patients compared with healthy controls (Supplemental Figure 1B), suggesting that the mutant alleles were not degraded. Sequencing the cDNA of the patients who are heterozygotes for either c.58_62del, c.98delT or c.162+2T>C variants detected equal amplitudes of the mutant and WT alleles (Supplemental Figure 1, A and C), corroboratively indicating escape from nonsense-mediated mRNA decay (NMD) of the mutant transcripts.

Next, we examined the GLTP protein expression by Western blotting analysis using primary keratinocytes isolated from the skin lesions from P1, P2, and P3. *GLTP* produces a 5-exon transcript encoding a 209-amino-acid protein (Supplemental Figure 1D). An antibody raised against an N-terminal peptide encompassing amino acids 2–51 of the GLTP protein was employed. As shown in Figure 2B, an approximately 23 kDa band corresponding to the full-length GLTP protein was detected in 4 normal control samples, whereas no specific GLTP signal, either the full-length or any truncated forms, was detected in samples from P1, P2, and P3. Immunofluorescence analysis of GLTP on normal human skin section demonstrated a strong expression in the differentiated suprabasal layers of the epidermis, whereas the GLTP protein expression was barely or weakly detected in the *GLTP*-nEDD patients’ skin lesions (Figure 2C).

To ascertain whether the lack of truncated protein detection was due to actual loss of expression or limited antibody recognition of incomplete epitopes, we conducted an overexpression assay. HEK293 cells were transfected with N-terminal FLAG-tagged WT GLTP and 2 patient-derived frameshift mutants, p.T20Afs*137 and p.F33Sfs*17. Western blotting with the anti-GLTP antibody detected a clear band at approximately 27 kDa in the WT sample, consistent with the full-length FLAG-tagged GLTP protein. A similar band was also detected by using the anti-FLAG antibody, confirming specificity of the GLTP antibody (Supplemental Figure 2A). Notably, no truncated protein in the cells expressing either GLTP frameshift mutant was detected by using the anti-FLAG antibody (Supplemental Figure 2A), suggesting the truncated proteins might undergo translational repression or posttranslational degradation. Although we cannot rule out the production of truncated proteins below detection limit, any such proteins are incapable of forming the hydrophobic pocket structure required for encapsulating glycosphingolipid hydrocarbon chains (Supplemental Figure 2B) (26, 27).

### Gltp-knockout mice show disrupted skin barriers and neonatal lethality.

**Figure 3:**
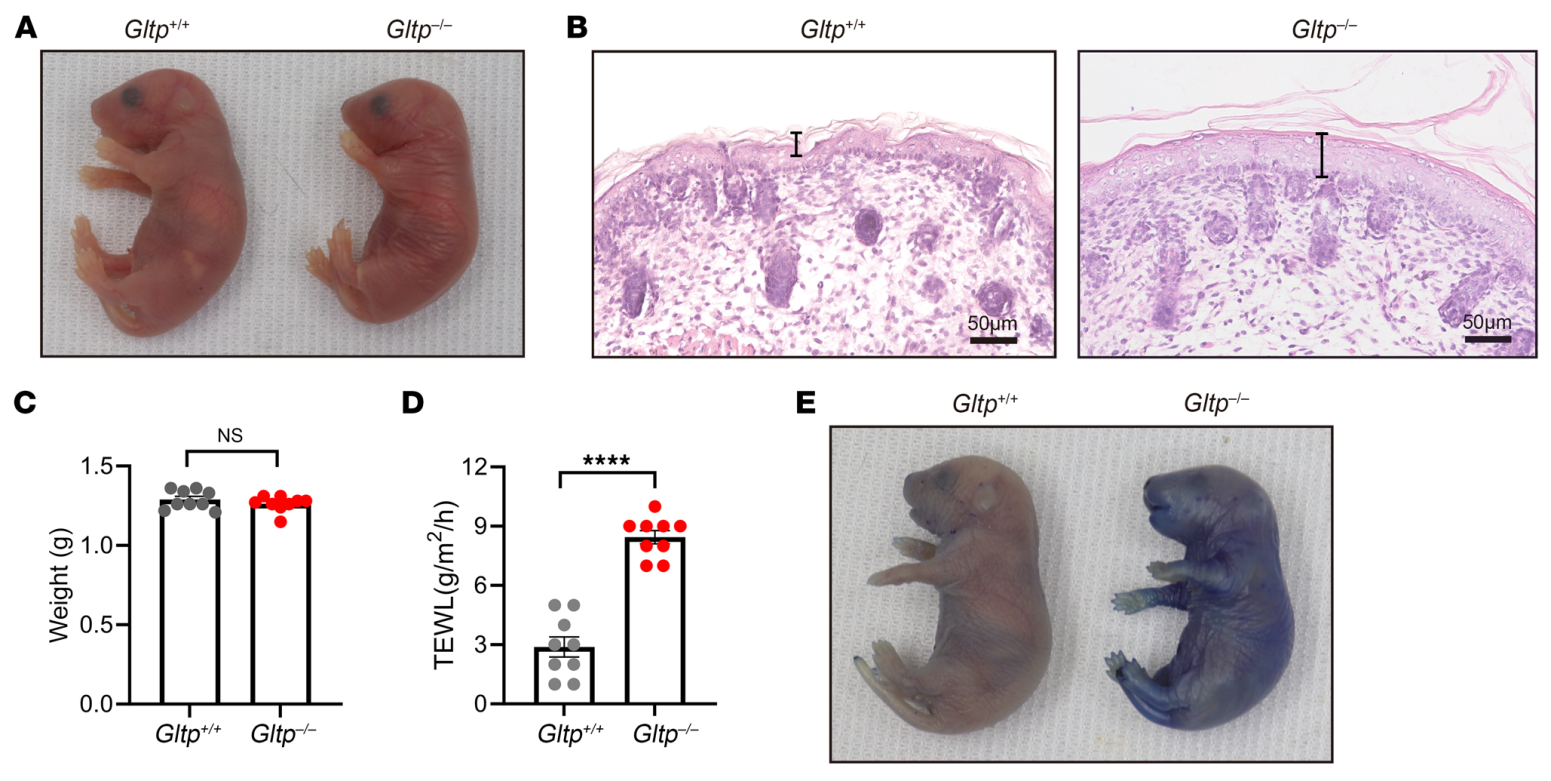
Phenotypic characterization of *Gltp*-deficient mice. (**A**) Gross appearance of *Gltp*^+/+^ and *Gltp^–/–^* neonates at 1 hour after birth. (**B**) H&E staining on dorsal skin sections of newborn *Gltp*^+/+^ and *Gltp^–/–^* mice. (**C**) Body weights of newborn *Gltp*^+/+^ (*n* = 9) and *Gltp^–/–^* mice (*n* = 9). (**D**) Transepidermal water loss (TEWL) measured on the dorsal skin of newborn *Gltp*^+/+^ (*n* = 9) and *Gltp^–/–^* mice (*n* = 9). (**E**) Skin permeability barrier function of newborn *Gltp*^+/+^ and *Gltp^–/–^* mice assayed by toluidine blue staining. Data represent the mean ± SEM. NS, not significant, ****P < 0.0001 (Student’s *t* test).

To gain deeper insight into the GLTP function in vivo, we generated global *Gltp* knockout (*Gltp^–/–^*) mice by deleting exons 2–4 of the *Gltp* gene using CRISPR-Cas9 technique. Although the *Gltp* mRNA was not degraded, we still failed to detect the truncated GLTP protein in the *Gltp^–/–^* mouse epidermis by Western blot and immunofluorescence analysis (Supplemental Figure 3, A–C). Offspring of the heterozygote intercrosses were born at an expected ratio. Consistent with the patients with *GLTP*-nEDD, the *Gltp^–/–^* mouse pups demonstrated dry and wrinkled skin with waxy appearance (Figure 3A) and died within 96 hours after birth, while the heterozygous mice (*Gltp^+/–^*) did not exhibit any remarkable abnormalities in skin appearance and were indistinguishable from the WT pups (*Gltp^+/+^*). Histopathologically, *Gltp^–/–^* skin showed marked hyperkeratosis and acanthosis (Figure 3B). Although *Gltp^–/–^* newborn mice showed no significant weight loss at birth compared with their control littermates (Figure 3C), transepidermal water loss (TEWL) was markedly higher in the *Gltp^–/–^* newborns (Figure 3D), suggesting a severe defect in the inside-out barrier of the epidermis. Furthermore, we also assessed the outside-in permeability barrier using a toluidine blue exclusion assay, in which *Gltp^+/+^* mice effectively excluded the dye, whereas *Gltp^–/–^* mice showed notable dye penetration into the skin (Figure 3E). Together, these results demonstrate that *Gltp^–/–^* mice presented with severe defects in skin barrier function, resulting in increased water loss and neonatal lethality. These findings highlight the critical role of GLTP in maintaining the skin permeability barrier.

### Aberrant vacuole accumulation and defective LB secretion in GLTP-deficient epidermis.

**Figure 4:**
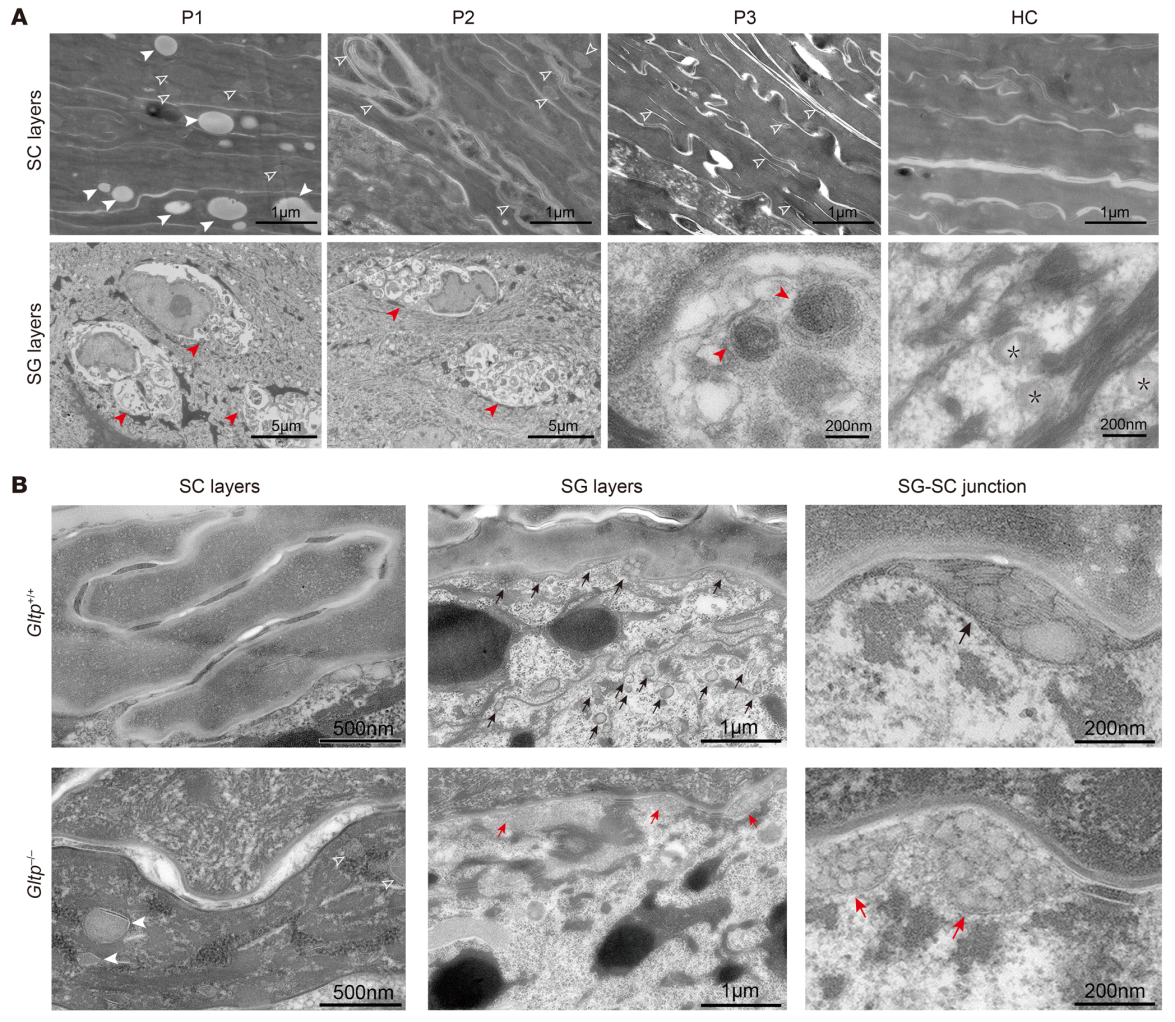
Ultrastructural changes in the epidermis of *GLTP*-nEDD patients and the *Gltp*-deficient mice. (**A**) Transmission electron microscopy (TEM) analysis of epidermal ultrastructure in patient (P) skin biopsies and a healthy control (HC). Representative images reveal distinct pathological features in patient corneocytes, including numerous vacuoles (solid white arrowheads) and curved membrane structures (hollow white arrowheads). Keratinocytes within the stratum granulosum exhibit irregular perinuclear accumulations of abnormal membranous and vesicular material (red arrowheads), accompanied by reduced lamellar body counts and disrupted lamellar organization. In contrast, healthy human epidermis demonstrates homogeneous, electron-dense, amorphous keratin substance in the stratum corneum and abundant normally structured lamellar bodies (black asterisks) in the granular layer. (**B**) TEM of murine epidermis indicates intact intercellular lipid lamellae in corneocytes layers of *Gltp*^+/+^ epidermis but absent in the *Gltp^–/–^* epidermis. *Gltp^–/–^* epidermis displayed numerous vacuoles in corneocytes (solid white arrowheads), curved membrane structures (hollow white arrowheads). In the stratum granulosum, lamellar bodies (red arrows) were reduced in number and display disrupted organization in *Gltp^–/–^* mice, contrasting with abundant well-structured lamellar bodies in *Gltp*^+/+^ epidermis (black arrows). SC, stratum corneum; SG, stratum granulosum.

To further characterize the morphological changes underlying the skin barrier defects, we performed ultrastructural analysis of epidermis in both patient skin biopsies and *Gltp^–/–^* murine models using transmission electron microscopy. The result revealed multiple perinuclear cystic vacuoles accompanied by extensive immature curved membrane structures in the cytoplasmic compartment in SG keratinocytes (Figure 4A), which was assumed to correlate with the cytoplasmic vacuoles observed in SG layer in the histological findings (Figure 1C). Moreover, LBs were absent or barely observed in the SC of all the patients in contrast with numerous LBs in normal control (Figure 4A). Additionally, numerous vacuoles and curved membrane structures were observed in the patients’ corneocytes. Taken together, these ultrastructural findings might suggest defective intracellular trafficking of lipids in the patients with GLTP defects.

We further examined the epidermal ultrastructure in the *Gltp*-knockout mice. In WT mice, well-organized, multilayered intercorneocyte lipid lamellae were observed in the SC. In contrast, remarkable disorganization of extracellular lipid lamellae was observed in the *Gltp^–/–^* epidermis (Figure 4B). Additionally, vacuoles with lipid-like inclusions were trapped within corneocytes of *Gltp^–/–^* mice (Figure 4B). In contrast with the multiple regularly structured LBs in *Gltp^+/+^* mice, the LBs of *Gltp^–/–^* granular cells lack typical lamellar structures. Despite the structural defects, the fusion of LBs with the granular cell membrane appeared unaffected in the *Gltp^–/–^* mice (Figure 4B). Collectively, the ultrastructural abnormalities in *Gltp^–/–^* mice suggested defective LB formation and disorganized epidermal lipid lamellae.

### Disrupted GlcCer spatial distribution in GLTP-deficient epidermis.

**Figure 5:**
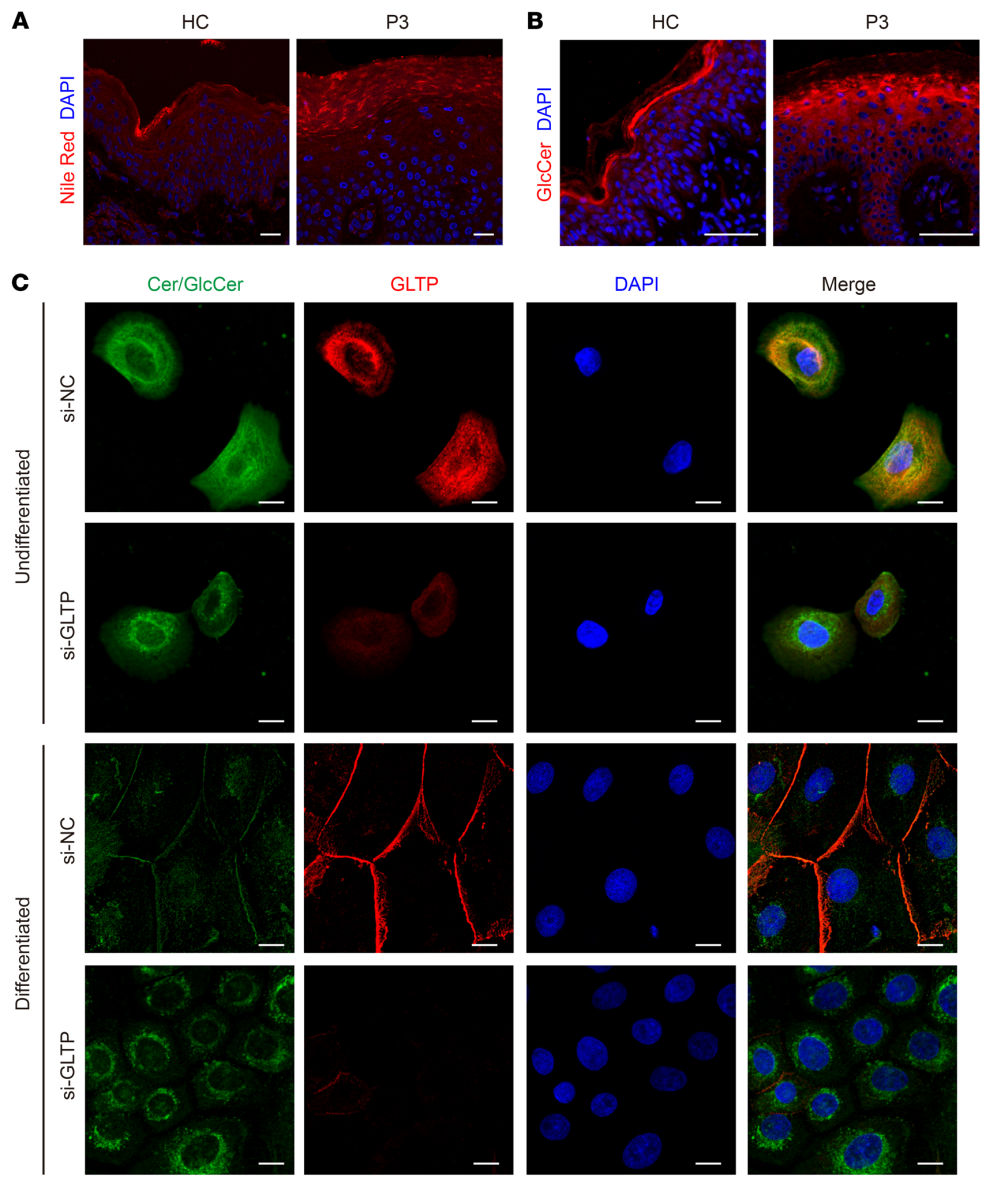
Aberrant glucosylceramide transport in *GLTP*-deficient differentiated keratinocytes. (**A**) Nile Red staining shows dot-like aggregates in the corneocytes of patient 3 (P3) compared with the continuous linear lipid structures observed in the healthy controls (HC). Scale bars: 20 μm. (**B**) Immunofluorescence of glucosylceramide (GlcCer) in the frozen skin section reveals increased GlcCer accumulation within the SG of the patient, in contrast to the presence in SC layer of the HC section. Scale bars: 50 μm. (**C**) Double immunofluorescent staining of GLTP and ceramide in primary human keratinocytes. Under undifferentiated condition, both GLTP and GlcCer/ceramide show cytoplasmic localization. GlcCer/ceramide immunocytochemistry demonstrated congested patterns of ceramide in undifferentiated si-*GLTP* keratinocytes. Change in localization of GLTP from cytoplasmic appearance to a both membranous and cytoplasmic appearance in response to Ca^2+^-induced differentiation. In contrast with downregulation and cell membranous distribution of ceramide in the si-NC differentiated keratinocytes, GlcCer/ceramide demonstrated accumulation and congested patterns of ceramide in differentiated si-*GLTP* keratinocytes. Scale bars: 10 μm.

Since GLTP has been demonstrated to facilitate the transport of various glycosphingolipids in vitro (28), we next examined whether GLTP deficiency disrupted epidermal lipids. To assess potential alterations in cutaneous lipid distribution, we performed Nile Red fluorescence staining on the epidermis from both patients with *GLTP*-nEDD and *Gltp^–/–^* mice. This solvatochromic lipophilic dye exhibits a polarity-dependent red-shift in its emission spectrum (29). In healthy controls, the SC layer exhibited highly organized stacks of red fluorescence in a weave-like distribution, whereas the patients displayed discontinuous, dot-like aggregates in the SC (Figure 5A), indicating aberrant accumulation of polar lipids. Since GlcCer is the most abundant glycosphingolipid in the epidermis (30), we next explored the GlcCer distribution in the skin epidermis from the patient with *GLTP*-nEDD and a healthy control using a GlcCer antibody. We found that GlcCer is abundantly detected in the outmost SG layer and weakly present in the SC layers of the healthy epidermis. In contrast, in the patients’ skin, diffuse distribution of GlcCer was identified throughout SG layers with a prominent cytoplasmic pattern, while it was nearly absent in the SC layer (Figure 5B).

We further examined the lipid distribution in the murine model and observed similar changes in the *Gltp^–/–^* mice. Specifically, the SC layer of the *Gltp^+/+^* mice exhibited continuous, highly organized stacks of lipid multilayers, while the SG layer showed evenly distributed, granular-like red fluorescent Nile Red aggregates. In contrast, *Gltp^–/–^* epidermis displayed disordered, dot-like and discontinuous linear-like lipid aggregates along with decreased granular lipid accumulation in the SG layer (Supplemental Figure 3D). Consistently, immunofluorescence staining for GlcCer in skin sections of *Gltp^–/–^* newborn mice revealed remarkably reduced fluorescent signals in SC, in contrast with the continuous GlcCer staining in both SG and SC layers in *Gltp^+/+^* epidermis (Supplemental Figure 3E).

### GLTP deficiency impaired GlcCer transport during keratinocyte differentiation.

To test whether GLTP is required for GlcCer transport, we conducted double-immunofluorescence staining of both GLTP and GlcCer in the cultured primary keratinocytes with GLTP deficiency. *GLTP* was knocked down using specific siRNA against GLTP (si-*GLTP*) with an efficiency of approximately 90% at the mRNA and protein level (Supplemental Figure 4, A and B). Since the anti-GlcCer antibody performed poorly in immunofluorescence staining on cells, we used an alternative anti-GlcCer/ceramide antibody (Enzo Life Sciences, MID15B4), which cannot distinguish between GlcCer and ceramides. In undifferentiated primary keratinocytes, GLTP showed a diffuse cytoplasmic distribution in scramble siRNA-treated (si-NC) keratinocytes, and the GlcCer/ceramide colocalized with the GLTP protein (Figure 5C). However, when GLTP was knocked down, GlcCer/ceramide exhibited perinuclear aggregation in the keratinocytes (Figure 5C), suggesting impaired trafficking. As GLTP is predominantly expressed in the terminally differentiated epidermal keratinocytes, we further examined the GlcCer/ceramide distribution in differentiated keratinocytes that were induced by elevated Ca^2+^ culture for 3 days. Strikingly, GLTP was distributed almost exclusively on the plasma membrane in the si-NC keratinocytes (Figure 5C). Meanwhile, in si-NC–differentiated keratinocytes, the GlcCer/ceramide fluorescence intensity was markedly attenuated compared with that of the si-NC undifferentiated keratinocytes, with a proportion of remaining signal localized to the plasma membrane (Figure 5C), a pattern consistent with previous literature reports (31), leading to a speculation that they have been transported to the extracellular space. In contrast, the si-*GLTP* keratinocytes demonstrated distinct punctate accumulation of GlcCer/ceramide in the perinuclear region upon differentiation (Figure 5C). Since GLTP is known to transport glycosphingolipids rather than ceramides (32), our results suggest that GLTP is involved in facilitating GlcCer transport to the extracellular space during keratinocyte differentiation.

### Epidermal lipid composition was altered in the Gltp^–/–^ mouse epidermis.

**Figure 6:**
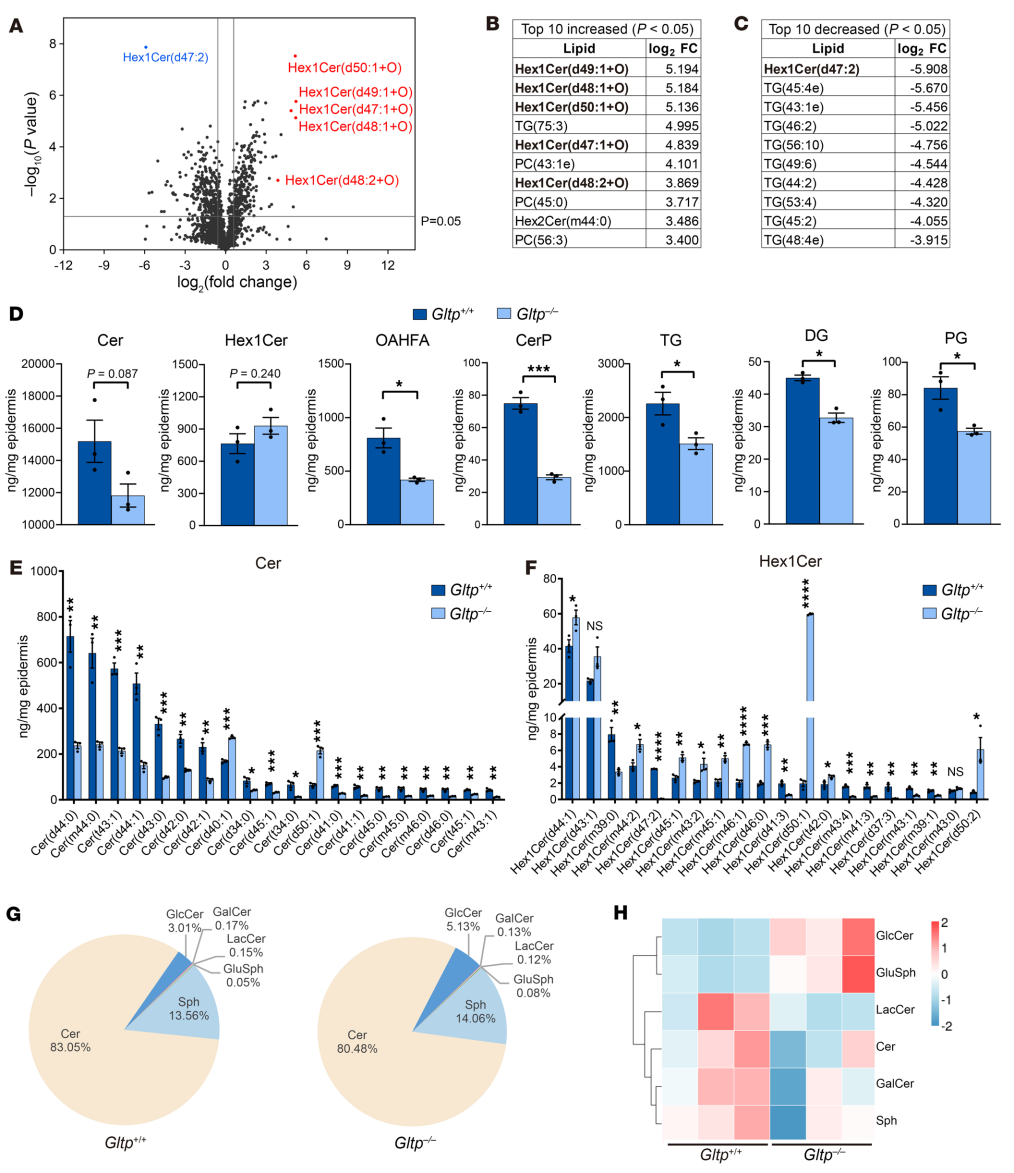
Epidermal lipid composition was altered in *Gltp^–/–^* mice. (**A**) Volcano plot of lipidomics depicting differentially altered lipid species in newborn *Gltp^–/–^* (*n* = 3) versus *Gltp^+/+^* mice (*n* = 3). Red and blue points denote upregulated and downregulated monohexosylceramides respectively, which are further highlighted in **B** and **C**. (**B** and **C**) Top 10 increased (**B**) or decreased (**C**) lipid species in *Gltp^–/–^* (*n* = 3) compared with *Gltp^+/+^* (*n* = 3) mice. Monohexosylceramides are highlighted in bold. (**D**) Quantitative profiling of 7 major lipid classes in the epidermis of *Gltp^+/+^* (*n* = 3) and *Gltp^–/–^* (*n* = 3) neonatal mice. (**E** and **F**) Comparative quantification of the top 20 monohexosylceramides and ceramides (ranked by abundance in *Gltp^+/+^* mice) in *Gltp^+/+^* (*n* = 3) and *Gltp^–/–^* (*n* = 3) newborn mouse epidermis. (**G** and **H**) Targeted sphingolipidomic profiling and analyses of newborn *Gltp^+/+^* (*n* = 3) and *Gltp^–/–^* mice (*n* = 3) epidermis. The pie chart depicts the relative abundance of major sphingolipid subclasses (**G**). (**H**) Heatmap showing the abundance of 6 major sphingolipid subclasses. The color gradient (blue to red) indicates lower to higher concentration. Rows represent sphingolipid subclasses, and columns represent samples/groups. Cer, ceramide; Hex1Cer, monohexosylceramide; OAHFA, (O-acyl)-1-hydroxy fatty acid; CerP, ceramide phosphate; TG, triglyceride; DG, diacylglycerol; PG, phosphatidylglycerol; GalCer, galactosylceramide; LacCer, lactosylceramide; Sph, sphingosine; GluSph, glucosylsphingosine. Data represent the mean ± SEM. *P < 0.05, **P < 0.01, ***P < 0.001, ****P < 0.0001 (Student’s *t* test).

To further investigate the impact of GLTP deficiency on the regulation of endogenous epidermal lipid composition, we analyzed epidermal lipids from newborn *Gltp^+/+^* and *Gltp^–/–^* mice using quantitative LC-MS/MS analysis. Lipids extracted from the epidermis of *Gltp^+/+^* (*n* = 3) and *Gltp^–/–^* (*n* = 3) mice at postnatal day 0 were analyzed. The volcano plot (Figure 6A) highlights lipid species differentially abundant between *Gltp^–/–^* compared with *Gltp^+/+^* mice. The top 10 increased and decreased species are detailed in Figure 6, B and C, respectively. Notably, monohexosylceramides (mainly GlcCer and galactosylceramide) constituted the majority of the increased lipids. Global lipidomic profiling of neonatal epidermis consistently showed a decrease in total ceramides and a relative increase in total monohexosylceramides in *Gltp^–/–^* neonatal mice compared with *Gltp^+/+^* mice, though statistically nonsignificant (*P* = 0.24) (Figure 6D). Moreover, marked reductions in multiple lipid classes, including (O-acyl)-1-hydroxy fatty acid, ceramide phosphate, triglycerides, diglycerides, and phosphatidylglycerol were observed in the *Gltp^–/–^* neonatal mice (Figure 6D). Further subclass analysis focused on ceramide species demonstrated a pronounced decrease in most ceramides (Figure 6E), while most monohexosylceramides showed significant accumulation in the *Gltp^–/–^* mice (Figure 6F). Since monohexosylceramides comprise both glucose (GlcCer) and galactose (galactosylceramide) as the carbohydrate component (30, 33), we further performed targeted sphingolipidomics to determine the specific contributor. This analysis revealed that GlcCer is the predominant monohexosylceramide in *Gltp^+/+^* epidermis, with its abundance nearly 20-fold higher than that of galactosylceramide (3.01% versus 0.17%). Moreover, within the monohexosylceramides, only GlcCer showed marked upregulation in the *Gltp^–/–^* mice (Figure 6, G and H). Together, these findings indicate that GlcCer is not only the major epidermal species but also the primary contributor to the observed increase in total monohexosylceramides.

### GLTP knockdown promotes keratinocyte proliferation and disturbs terminal differentiation.

**Figure 7:**
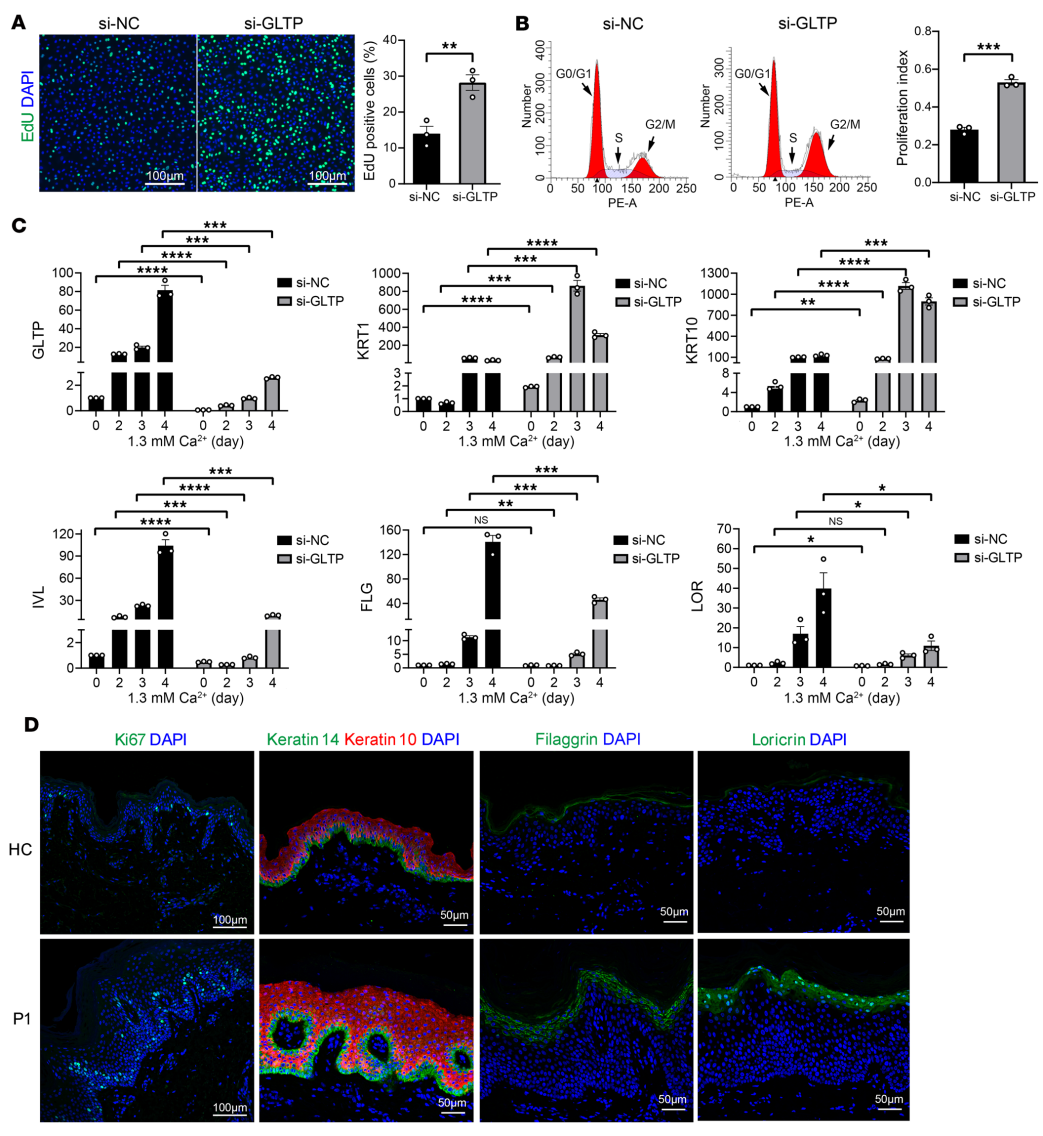
Knockdown of GLTP promoted keratinocyte proliferation and delayed terminal differentiation. (**A**) EdU staining shows enhanced proliferation in si-*GLTP* keratinocytes. (**B**) Flow Cytometry shows a higher percentage of cells in the S and G2/M phases in si-*GLTP* keratinocytes. (**C**) RT-qPCR profiling of differentiation markers in si-*GLTP* versus si-NC–transfected keratinocytes during Ca^2+^-induced differentiation. (**D**) Immunofluorescence of proliferation or keratinocyte differentiation markers in frozen (for keratin 14 and keratin 10) or paraffin-embedded (for Ki67, filaggrin and loricrin) skin sections. *n* = 3, ± SEM; *P < 0.05, **P < 0.01, ***P < 0.001, ****P < 0.0001 (Unpaired Student’s *t* test used in **A**–**C**; multiple comparisons were corrected using Benjamini, Krieger, and Yekutieli false discovery approaches).

As both GLTP and GlcCer have been implicated in their roles in keratinocyte differentiation (16), we silenced GLTP in human primary keratinocytes to assess the functional impact of GLTP deficiency on keratinocyte proliferation and differentiation. Firstly, we analyzed keratinocyte proliferation by EdU staining and flow cytometry cell-cycle experiments. We observed a significant increase in the percentage of EdU-positive cells in cells with downregulated GLTP compared with control (Figure 7A). Moreover, cell-cycle analysis showed that knock down of GLTP resulted in a significant increase in the proliferation index of keratinocytes (Figure 7B). These findings suggested that GLTP deficiency enhances the proliferation of keratinocytes.

Next, we assessed calcium-induced keratinocyte differentiation under GLTP knockdown conditions. Notably, we observed an upregulation of *GLTP* mRNA expression as well as several differentiation-related markers (*KRT1, KRT10*, *FLG*, *IVL*, and *LOR*) during differentiation in si-NC keratinocytes (Figure 7C), further confirming that the GLTP expression is positively correlated with keratinocyte differentiation. In contrast, in si-*GLTP* keratinocytes, the late differentiation markers *FLG*, *IVL*, and *LOR* in the si-*GLTP* cells did not exhibit pronounced upregulation as seen in the controls, while *KRT1* and *KRT10*, the early/mid-differentiation marker, were significantly more upregulated than in controls (Figure 7C). These findings suggested that GLTP deficiency impairs terminal differentiation of keratinocytes.

Consistently, immunostaining for the cell proliferation marker Ki67 revealed a remarkedly increased number of proliferating cells in patient skin lesions, consistent with the hyperproliferative phenotype (Figure 7D). Next, we assessed epidermal differentiation by immunostaining with antibodies to keratin 14 (a marker of basal keratinocytes) and keratin 10 (a marker of suprabasal keratinocyte differentiation). The patient exhibited a normal keratin 14/ keratin 10 differentiation switch. Nevertheless, keratin 10 expression was markedly broader and more intense in the individual, suggesting enhanced early/mid keratinocyte differentiation. Filaggrin is primarily localized in the keratohyaline granules of the SG, where profilaggrin is proteolytically cleaved into filaggrin monomers, which crosslink keratin intermediate filaments through disulfide bonds during cornification. In healthy control skin, filaggrin was predominantly found in the upper SG and SC, while, in the patient’s epidermis, it appeared in strong expression observed in the expanded SG. Loricrin, a major component of the cornified envelope, is typically expressed in the later stages of cornified envelope formation. In the healthy control, loricrin expression was mainly confined to the SG/SC interface, while it exhibited strong cytosolic staining and diffuse nuclear mislocalization in the patients’ SG keratinocytes. These findings suggest a delay or defect in the processing and localization of profilaggrin and loricrin in patients. Overall, these results implicate disorganized keratinocyte terminal differentiation and hyperproliferative features in the patients. Immunofluorescence staining of differentiation markers keratin 10, filaggrin, and loricrin of *Gltp^–/–^* mice showed similar changes to that of the patients (Supplemental Figure 5).

### GLTP silencing dysregulates gene networks controlling keratinocyte differentiation, skin barrier integrity, and autophagy balance.

**Figure 8:**
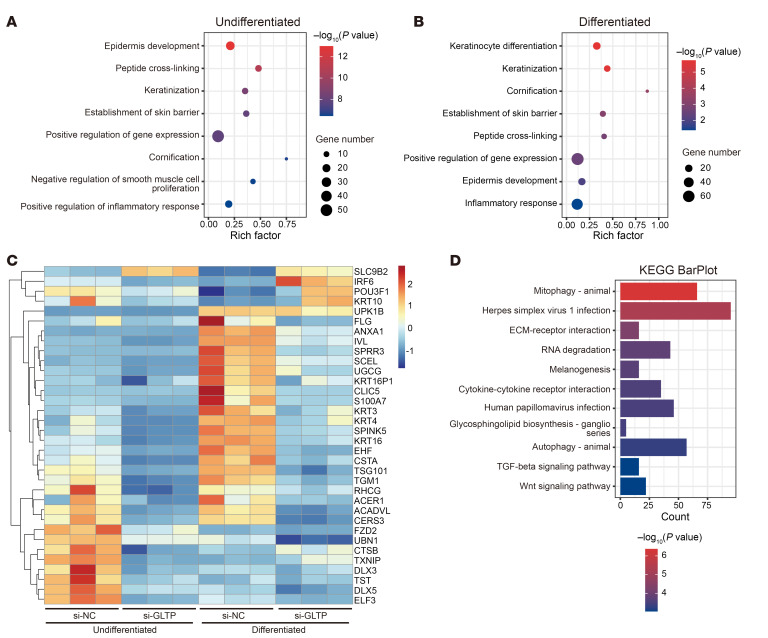
Deregulated genes associated with keratinocyte differentiation and establishment of skin barrier in GLTP-knockdown keratinocytes. (**A** and **B**) Gene Ontology (GO) enrichment for significantly differentially expressed genes (DEGs) identified from RNA-seq analysis in undifferentiated (**A**) and differentiated conditions (**B**). (**C**) Heatmap showing keratinocyte differentiation–related genes enriched in the GO Biological Processes (BP). (**D**) Bar plot of significantly enriched KEGG terms for the DEGs.

To explore the molecular mechanism underlying the abnormal terminal differentiation caused by *GLTP* deficiency, we conducted transcriptomic analysis on si-NC and si-*GLTP* keratinocytes under basal and Ca^2+^-induced differentiated conditions. We identified 1,645 and 2,732 differentially expressed genes (DEGs) in si-*GLTP* compared with si-NC keratinocytes under undifferentiated and differentiated conditions, respectively. Gene ontology enrichment analysis of DEGs revealed significant enrichment of genes associated with cornification, epidermis development, and establishment of skin barrier (Figure 8, A and B). Notably, although the si-NC group demonstrated a complete transition to differentiation status upon high-calcium induction, the si-*GLTP* keratinocytes showed minimal differentiation progression. Specifically, several key genes involved in the terminal differentiation processes in keratinocytes, including filaggrin (*FLG*), involucrin (*IVL*), and transglutaminase-1 (*TGM1*), did not show significant upregulation under differentiation condition, with only the middifferentiation marker *KRT10* slightly upregulated in si-*GLTP* cells (Figure 8C). These results collectively indicate that *GLTP* knockdown specifically impeded the terminal differentiation process. Additionally, the transcriptomic analysis revealed significant downregulation of several ceramide synthesis-related genes that are critical for epidermal lipid barrier establishment, including *UGCG* (encoding GlcCer synthase), *ACER1* (alkaline ceramidase 1), and *CERS3* (ceramide synthase 3) in si-*GLTP* keratinocytes compared with the si-NC keratinocytes under differentiation condition (Figure 8C). Intriguingly, we also noticed pronounced enrichment in autophagy and mitophagy pathways through KEGG pathway analysis of DEGs in differentiated keratinocytes (Figure 8D).

### Impaired autophagy flux in GLTP-deficient epidermis.

**Figure 9:**
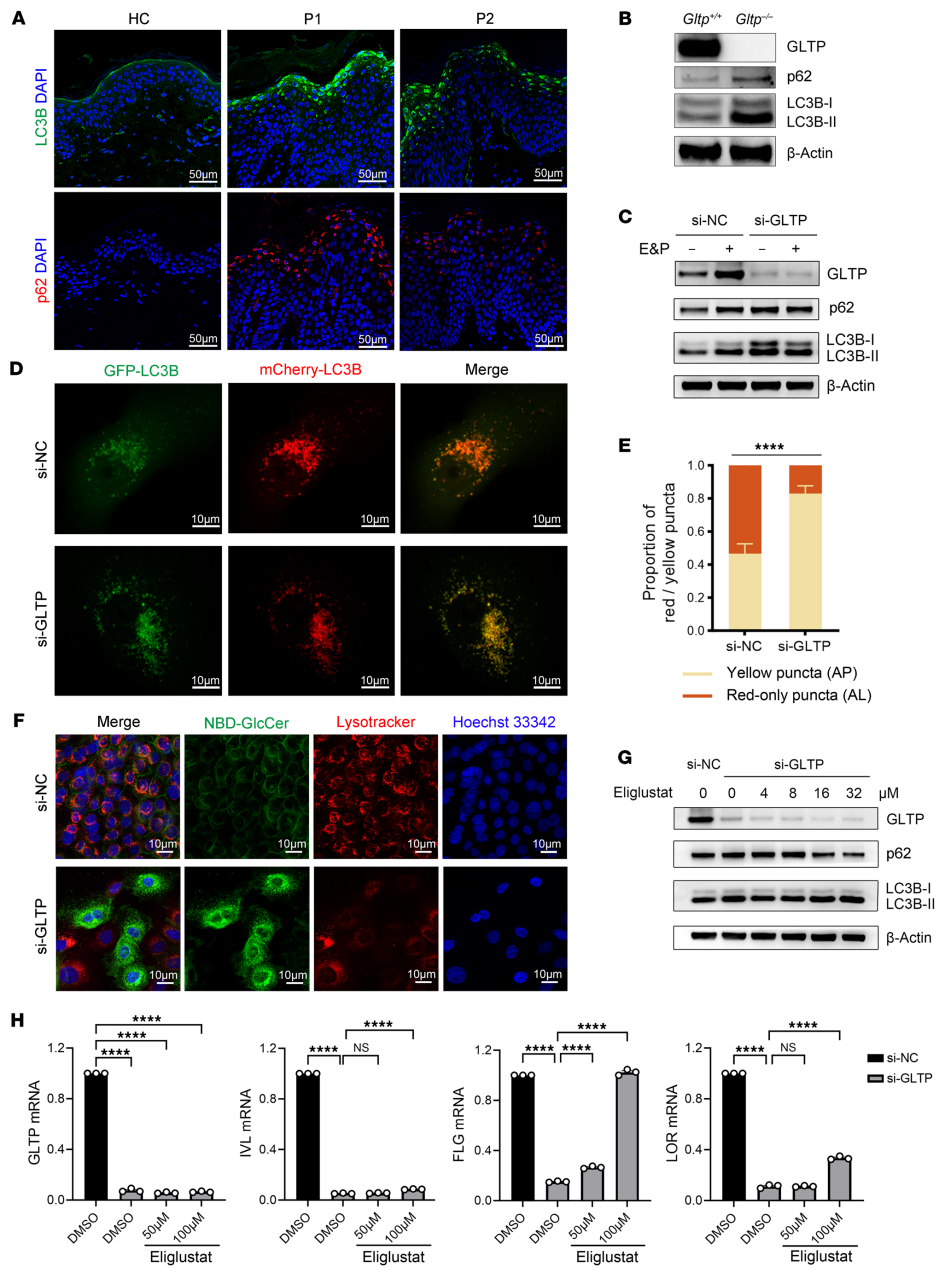
GLTP deficiency impaired autophagy flux via lysosomal GlcCer accumulation in keratinocytes. (**A**) Immunofluorescence staining of LC3B and p62 in the epidermis of patients (P1, P2) and HC. Scale bars: 50 μm. (**B**) Western blot of LC3B and p62 protein expression in the epidermis from *Gltp^+/+^* and *Gltp^–/–^* mouse newborns. (**C**) Expression levels of autophagic markers LC3B and p62 in si-*GLTP* and si-NC keratinocytes treated with E64d & Pepstatin A (E&P). (**D**) Representative images of keratinocytes infected with adenovirus Ad-mCherry-GFP-LC3B. Scale bars: 10 μm. (**E**) Quantification analysis of autophagosomes (AP, yellow puncta in merged images) and autolysosomes (AL, red-only puncta in merged images) in keratinocytes. Bars represent the mean ± SEM from 3 independent experiments (40–50 cells in total per group). (**F**) Distribution of C6-NBD-GlcCer (green), a fluorescent GlcCer analog, in differentiated keratinocytes with lysotracker (red) and Hoechst nuclear stain (blue). Scale bars: 10 μm. (**G**) Expression of autophagic markers LC3B and p62 in si-*GLTP* keratinocytes treated with varying doses of Eliglustat. (**H**) RT-qPCR profiling of differentiation markers *IVL*, *FLG*, and *LOR* in Ca^2+^-induced differentiation keratinocytes treated with Eliglustat. *n* = 3, ± SEM; ****P < 0.0001 (1-way ANOVA with Dunnett’s multiple comparison test).

Autophagy, a constitutively active process in the SG layer, drives senescent keratinocytes into cell death in the process of terminal differentiation (34–36). Its impairment has been implicated in the pathogenesis of parakeratotic disorders, such as psoriasis (37, 38). To determine the autophagic status, we performed immunofluorescence analysis of 2 well-established autophagy markers, LC3B and p62, in skin biopsies from patients and healthy controls. During autophagy, LC3B-I is converted to its lipidated form LC3B-II, which is subsequently recruited to autophagosomal membranes (39), thus, the LC3B-II/I ratio is generally used as a well-established biochemical indicator of autophagosome formation. p62 is an autophagic receptor that binds directly with LC3B to mediate the selective degradation of ubiquitinated substrates, thus serving as a complementary indicator of autophagic degradation activity (40). In control biopsy, both LC3B and p62 displayed minimal expression and were confined to the SG, consistent with a previous report (Figure 9A) (41). Notably, the patient skin biopsies demonstrated robust LC3B and p62 expression, with intense perinuclear staining throughout the SG and upper SG layers (Figure 9A), suggesting aberrant autophagy. Consistently, Western blot analysis also showed increased LC3B-II and p62 expression in neonatal *Gltp^–/–^* mouse epidermis (Figure 9B), which is an indicator of impaired autophagic flux likely caused by disturbed autophagic degradation.

### GlcCer accumulation perturbed lysosomal functionality, resulting in autophagy arrest.

As a crucial step of autophagy process, the cytoplasmic components delivered by the autophagosomes are degraded by lysosomal hydrolases (42). To further confirm that GLTP deficiency impairs autophagic flux, we tested autophagic flux in GLTP-deficient differentiated primary human keratinocytes using lysosomal protease inhibitors E64d and pepstatin A (E&P). As shown in Figure 9C, the si-NC keratinocytes expressed low levels of LC3B-II and p62 under basal conditions and responded to E&P treatment as expected, with a marked accumulation of LC3B-II and p62. In contrast, the si-*GLTP* keratinocytes showed elevated levels of both LC3B-II and p62 at basal levels compared with the si-NC group, but failed to increase further upon E&P treatment, which implied that the autophagic flux might be maximally impaired in these cells. These findings collectively suggest that GLTP deficiency leads to inhibited autophagic flux, with the defect likely occurring at the lysosomal degradation stage.

Next, we employed a dual-fluorescence pH-sensitive reporter AdPlus-mCherry-GFP-LC3B, which consists of a tandem fusion of the acid-insensitive mCherry and the acid-sensitive green fluorescent proteins (GFP). This established system enables precise monitoring of lysosome, a critical indicator of functional autophagic progression. Neutral autophagosomes exhibited dual-fluorescence labeling (yellow signal from mCherry and GFP colocalization), whereas acidified autolysosomes displayed selective mCherry fluorescence (red) due to pH-dependent GFP quenching in the acidic compartment (43). We observed that both si-NC and si-*GLTP*–transfected primary keratinocytes exhibited dual red and green fluorescent signals, while the si-*GLTP* group showed markedly more enhanced green puncta intensity, yielding a more yellowish merged fluorescence that indicated reduced GFP quenching (Figure 9D). Quantitative analysis demonstrated that, while si-NC controls contained approximately 50% red puncta (mature autolysosomes) versus 50% yellow puncta (autophagosomes), the si-*GLTP* transfected cells displayed significantly increased co-localization with approximately 80% yellow puncta (Figure 9E), indicating the impaired autophagic flux was associated with reduced lysosomal acidification.

To explore the underlying mechanism, we asked whether the trafficking defect of GlcCer contributed to the impaired lysosome function. We conducted dual-immunofluorescence analysis of GlcCer/ceramide and lysosome marker LAMP1 in fixed differentiated primary keratinocytes. The result revealed that GLTP deficiency led to marked perinuclear sequestration of GlcCer/ceramide, with partial colocalizing with the lysosome marker LAMP1, in contrast with the diffuse cytoplasmic staining with additional plasma membrane localization of GlcCer/ceramides in si-NC group (Supplemental Figure 6). To further assess lysosomal involvement, we performed live-cell imaging using LysoTracker Red DND (a pH-dependent dye for labelling lysosomes) as well as the fluorescent GlcCer analog C6-NBD-GlcCer. As demonstrated in Figure 9F, the C6-NBD-GlcCer displayed a predominant plasma membrane distribution in si-NC keratinocytes, while the si-*GLTP* cells exhibited pronounced perinuclear GlcCer accumulation. Strikingly, LysoTracker fluorescence intensity was markedly attenuated in si-*GLTP* keratinocytes, indicating compromised lysosomal acidification, which is likely a consequence of GlcCer overload.

### Inhibition of GlcCer synthase rescued the impaired autophagic flux and defective keratinocyte differentiation.

Eliglustat is a selective inhibitor of UDP-glucose ceramide glucosyltransferase, the rate-limiting enzyme in glycosphingolipid biosynthesis. We further tested whether this inhibitor could reverse the aberrant autophagy and the defective keratinocyte differentiation phenotypes through inhibiting GlcCer production in GLTP-deficient keratinocytes. We treated GLTP-deficient primary keratinocytes with Eliglustat during differentiation. Western blot demonstrated a dose-dependent restoration of autophagic flux, evidenced by a marked reduction in p62 levels at concentrations greater than or equal to 16 μM (Figure 9G). Intriguingly, LC3B-II exhibited biphasic dynamics: suppression at low doses (4–8 μM), suggesting enhanced autophagosome clearance, followed by accumulation at higher doses (16–32 μM), indicative of reactivated autophagosome formation.

We next evaluated keratinocyte differentiation by measuring the mRNA expression of several differentiation markers *KRT10*, *IVL*, *FLG*, and *LOR*, following treatment with Eliglustat at 50 and 100 μM, respectively, for 3 days. We observed a slight restoration of *FLG* expression at 50 μM of Eliglustat group, while 100 μM of Eliglustat resulted in a more robust increase in the expression of *FLG*, as well as a modest elevation of *IVL* and *LOR* (Figure 9H). Collectively, these results demonstrate that pharmacological inhibition of GlcCer synthesis by Eliglustat dose-dependently rescued both impaired autophagic flux and defective keratinocyte differentiation in GLTP-deficient keratinocytes.

## Discussion

At least 13 genes have been linked to autosomal recessive congenital ichthyosis. Exploration of the clinical effects of pathogenic variants in these causative genes could provide insights into the molecular mechanisms underlying skin barrier functions. Notably, disturbances of lipid metabolism represent a predominant pathogenic mechanism in ichthyosis. Currently identified ichthyosis-related genes contribute through several mechanisms including (a) lipid synthesis defects, exemplified by CERS3, ALOX12B, and PNPLA1 deficiencies that impair ceramide production (44–46); (b) disrupted lipid degradation, including STS deficiency causing excessive cholesterol sulfate accumulation (47), and ABHD5 deficiency resulting in defective triglyceride lipolysis and consequent aberrant deposition (48); (c) disrupted lipid transport, as demonstrated by ABCA12 deficiency, which compromises lipid trafficking, leading to abnormal GlcCer accumulation and reduced SC ceramide levels (8).

We herein identified a new ichthyosis gene involved in lipid transport. In contrast to the severe, even lethal phenotype caused by *ABCA12* variants, biallelic loss-of-function variants in *GLTP* lead to a mild ichthyosis phenotype characterized by xerosis, scaling, and defective skin barrier properties. This phenotype was closely recapitulated in the *Gltp^–/–^* mice, which included hallmark features of congenital ichthyosis such as significantly elevated TEWL, defective cutaneous dye exclusion, and neonatal lethality secondary to severe permeability barrier dysfunction. Although the *Gltp^–/–^* mice exhibit neonatal lethality, in contrast with the otherwise healthy phenotype of the patients with *GLTP*-nEDD, such disparity is not uncommon in other genetic knockout models of autosomal recessive congenital ichthyosis (e.g., *TGM1*, *ABCA12*, *PNPLA1*, *CERS3*, *NIPAL4*, and *SDR9C7*) (49–54). We speculate that it may be attributed to the profound physiological vulnerability of neonatal mice, characterized by thinner skin and an unfavorable surface area–to–volume ratio that predisposes them to rapid dehydration (51, 55, 56). Furthermore, only about 30% of the top skin-associated genes show transcriptional conservation between humans and mice (57), indicating substantial divergence in their epidermal transcriptional programs. Collectively, findings from patients with *GLTP* variants and the *Gltp^–/–^* mouse model establish loss of function in GLTP, a glycolipid transporter, as the cause of a previously undescribed form of nEDD.

Notably, the mutant *GLTP* transcripts showed no discernible degradation, indicating an escape from NMD. This observation aligns with the well-established rules for NMD escape mechanisms that mainly depend on premature termination codon (PTC) positioning (58). Particularly, both the c.58_62del variant and exon 2 skipping caused by c.162+2T>C variant generated PTCs in the last exon (exon 5), where NMD evasion occurs due to the absence of downstream exon junction complexes (EJCs). The c.98delT variant introduces a PTC in exon 2 positioned less than 50 nucleotides upstream of the downstream EJC, a configuration that prevents NMD through ribosome-mediated EJC during translation termination. Despite NMD evasion, we failed to detect the truncated GLTP proteins in either patient skin samples or in the extracts of cells overexpressing the mutant constructs, suggesting that the truncated proteins might undergo translational repression or posttranslational degradation. Previous studies have reported that some transcripts containing PTCs, which escape NMD might be subject to translational repression (59–61), representing a cellular protection mechanism against deleterious proteins.

We demonstrated that GLTP expression is correlated with keratinocyte differentiation and plays an indispensable role in GlcCer trafficking during skin terminal differentiation. Specifically, the in vitro experiments revealed that GLTP exhibited a distinct subcellular redistribution during keratinocyte differentiation, which shifted from a predominantly cytoplasmic localization in undifferentiated cells to a primarily cell-periphery pattern in differentiated cells. This redistribution was accompanied by a markedly diminished cytoplasmic GlcCer levels, with residual GlcCer showing preferential membrane localization, suggesting that GLTP participated in a certain step of GlcCer final extrusion in differentiated keratinocytes. These findings were further validated by GlcCer immunofluorescence in the patient skin samples and *Gltp^–/–^* mice, which demonstrated abnormal accumulation of GlcCer in the cytoplasm of the SG layer, contrasting with the normal physiological distribution pattern where GlcCer is predominantly localized to the outmost SG and SC layers. These observations collectively indicate that GLTP deficiency disrupted GlcCer transport from cytoplasmic SG keratinocytes to the SG-SC interface, the critical site for its conversion to ceramides. This is further supported by our lipidomic analysis, which revealed increased GlcCer levels accompanied by reduced ceramide content in the *Gltp^–/–^* mouse epidermal tissue.

Intriguingly, impaired GlcCer trafficking and defective LB formation were also observed in ABCA12-deficient keratinocytes. *ABCA12* is another causative gene for ichthyosis, where different mutations lead to varying disease severity. Severe ABCA12 deficiency due to biallelic loss-of-function variants causes harlequin ichthyosis, a lethal form of ichthyosis characterized by thick, armor-like plates of hyperkeratotic skin, along with severe ectropion, eclabium, and many life-threatening complications (8, 62). As an ATP-driven transmembrane lipid transporter, ABCA12 mediates the transport of diverse lipids into the inner side of LBs of the keratinocytes (8). Meanwhile, GLTP, which facilitates nonvesicular transport at membrane contact sites between Golgi and other organelles, has been detected in keratinocyte LB extracts (16), implicating its involvement in LB lipid transport. Our functional studies demonstrate that GLTP is essential for GlcCer transport, leading us to hypothesize that GLTP and ABCA12 may play complementary yet distinct roles in epidermal GlcCer transport. Given their distinct subcellular localizations and mechanistic differences, we propose a coordinated transport model in which GLTP mediates nonvesicular GlcCer transfer from trans-Golgi to the cytosolic aspect of LBs, while ABCA12 mediated translocation of GlcCer across the LB membrane into its lumen for subsequent secretion, a two-step process ensuring proper epidermal lipid barrier formation. Based on this theory, the phenotypic differences in ichthyosis caused by GLTP versus ABCA12 mutations can be mechanistically explained by their distinct roles in lipid transport. GLTP solely mediates the delivery of GlcCer from the trans-Golgi to the cytosolic leaflet of LBs, thus accounting for the relatively mild phenotypes observed even with biallelic GLTP defects. By contrast, ABCA12 functions as the essential and nonredundant transporter that flips various lipid species across the LB membrane into the lumen side. Severe ABCA12 deficiency disrupts this critical final step of lipid transport, and, consequently, leads to more severe forms of ichthyosis, such as harlequin ichthyosis. Nevertheless, further studies employing high-resolution spatial visualization techniques, such as immunoelectron microscopy or live-cell super-resolution imaging, may aid in clarifying the spatial relationship and functional interplay between GLTP and ABCA12 during lipid transport, thereby providing key insights into the proposed 2-step transport model.

As a lipid transfer protein, GLTP is well recognized as a nonvesicular lipid transporter (14). Several studies have suggested that GlcCer can reach the plasma membrane directly through a nonvesicular route in several cell types (10, 63). Notably, the Golgi apparatus undergoes extensive remodeling during epidermal keratinocyte differentiation, transitioning from compact to extensively spread and branched configurations (64). This structural dispersion likely positions the Golgi in close proximity not only to its derivative LBs but also to the plasma membrane. This spatial proximity potentially facilitates the formation of membrane contact sites, which serve as critical platforms for nonvesicular lipid transport (65, 66). Since GLTP efficiently transfers glycolipids between cytosolic membrane surfaces driven solely by concentration gradients and without organelle specificity, we cannot exclude the possibility that GLTP also directly mediates GlcCer transport from the Golgi to the plasma membrane. Apart from this, emerging evidence also suggests that GLTP may mediate vesicular trafficking by interacting with the ER membrane protein vesicle-associated membrane protein–associated protein-A (VAP-A) (67). It has been demonstrated that either GLTP knockout or disruption of its VAP-A binding in HeLa cells resulted in impaired vesicle transport between the ER and the plasma membrane. Interestingly, all mutations identified in this study are located within or upstream of residues 32–38, which form an FFAT (2 phenylalanines in an acidic tract) motif critical for VAP-A binding (68), leading to impaired interaction between GLTP and VAP-A. Therefore, further studies are warranted to investigate whether defective vesicular trafficking via VAP-A contributes to the pathogenesis of *GLTP*-nEDD.

We demonstrated that GLTP deficiency results in enhanced keratinocyte proliferation and defective differentiation, suggesting that the hyperkeratosis observed in patients resulted not only from compensatory barrier repair but from primary defects in epidermal homeostasis. Notably, *Abca12*-knockout mice exhibit not only a disrupted skin barrier but also impaired keratinocyte differentiation (69), although the mechanistic link between ABCA12 deficiency and differentiation defects remains unclear. These findings collectively underscore the essential role of GlcCer transfer for coordinating keratinocyte proliferation and differentiation. Our data indicate that the defective terminal differentiation is associated with the impaired autophagy, characterized by lysosome dysfunction and impaired autophagic flux. Autophagy is a highly active lysosome degradation process within the SG layer, where it continuously eliminates damaged organelles and proteins to sustain normal epidermal terminal differentiation (70). Autophagy dysfunction has been pathogenically linked to skin barrier dysfunction diseases such as atopic dermatitis (38). Here, we showed that lysosomal GlcCer accumulation in the differentiated keratinocytes affected lysosome function, which led to impaired autophagic flux. Additionally, our preliminary experiments showed that treatment with eliglustat rescued both autophagy impairment and differentiation defects in cultured cells, suggesting its promising therapeutic potential for this disorder.

In summary, we found that loss-of-function variants in *GLTP* lead to nEDD with impaired GlcCer transport. To the best of our knowledge, this is the first monogenic disorder in humans associated with variants in the *GLTP* gene. The study highlights the role of GLTP in epidermal lipid transport and its implications in regulating keratinocyte differentiation, suggesting it as a therapeutic target in nEDD.

## Methods

### Sex as a biological variable.

Sex was not considered as a biological variable in both human and animal experiments. Our study examined male and female humans and animals, and similar findings are reported for both sexes.

### Participants and sample collection.

Six unrelated patients with congenital ichthyosis were enrolled in this study. Skin biopsies were collected from ichthyosis lesions on the abdomen of P1, the lower back of P2, and the lower leg of P3.

### Genetic analysis.

Trio-based whole-exome sequencing (WES) was performed on genomic DNA from the peripheral blood of individuals P1, P2, and P3 and the parents of each patient by MyGenostics. In brief, exome capture and enrichment were carried out using the NimbleGen SeqCap EZ Exome Enrichment Kit v3.0 (Roche), followed by sequencing on the Illumina HiSeq 2000 platform. Candidate variants were confirmed via Sanger sequencing, and cosegregation analysis of genotype and phenotype was conducted among family members. The primers used are listed in Supplemental Table 1.

Whole genome sequencing in P5 and P6 was undertaken as part of the 100 000 Genomes Project (22).

### Cell culture.

Skin biopsies obtained from both patients and controls were enzymatically separated using dispase II solution (2.5 mg/mL, ThermoFisher Scientific, Cat# 17105041) overnight at 4°C. Then, the epidermis was dissociated into single-cell suspension by trypsin digestion for 5 minutes at 37°C. After neutralization, cells were pelleted and cultured in EpiLife medium (MEPI500CA, ThermoFisher Scientific) supplemented with human keratinocyte growth supplements (HKGS; S0015, ThermoFisher Scientific) at 37°C in an incubator with 5% CO_2_. Keratinocyte differentiation was induced by adding 1.3 mM CaCl_2_ to the EpiLife media (MEPICF500, ThermoFisher Scientific) supplemented with HKGS (S0015, ThermoFisher Scientific). HEK293 cells were purchased from ATCC and cultured in DMEM with 10% fetal bovine serum.

### RNA interference.

siRNA oligonucleotide duplexes targeting *GLTP* (si-*GLTP*, 5′-GCTGGATCGTGCAGAAGAT-3′) and scramble control siRNA (si-NC) used in this study were synthesized. Primary human keratinocytes were transfected with the indicated siRNA using Lipofectamine RNAiMAX (ThermoFisher Scientific) according to the manufacturer’s protocol.

### Plasmid constructs, mutagenesis, and transfection.

The pCMV-FLAG-GLTP plasmid expressing N-terminally FLAG-tagged human GLTP was constructed. The GLTP coding sequence was amplified from normal human keratinocyte cDNA and cloned using the ClonExpress II One Step Cloning Kit (C112, Vazyme). Site-directed mutagenesis was generated using the Mut Express II Rapid Mutagenesis Kit V2 (C214, Vazyme). All constructs were verified by sequencing. HEK 293 cells were transfected using Lipofectamine 3000 (L3000150, Thermo Fisher Scientific).

### RNA extraction and RT-qPCR.

Total RNA was extracted from human primary keratinocytes using the Quick-RNA Miniprep Kit (Goonie) according to the manufacturer’s protocol. First-strand cDNA synthesis was performed using the EVO M-MLV RT MIX Kit (AG11706, Agbio). qPCR was conducted using SYBR Green Pro Taq HS Premix (AG11701, Agbio) on a LightCycler 480II system (Roche). The *GAPDH* gene served as an endogenous control, and relative gene expression was calculated using the 2^–ΔΔCt^ method. Primer sequences for qPCR are provided in Supplemental Table 1.

### Protein extraction and Western blotting.

The cells were harvested and lysed in Pierce IP Lysis Buffer (87787, Thermo Fisher Scientific) containing protease inhibitor (Roche) according to the manufacturer’s instructions. The extracted protein was denatured, separated by SDS-PAGE electrophoresis and transferred to nitrocellulose membranes. Membranes were blocked with 5% milk for 1 hour at room temperature and incubated with the primary antibodies overnight at 4°C. The following primary antibodies were used for western blotting: GAPDH (1:1000, TA-08, ZSGB Biotech), GLTP (1:1000, Novus Biologicals, NBP2-31642), anti-LC3B (1:1000, Abcam, ab192890), anti-SQSTM1/p62 (1:1000, Abcam, ab91526), anti-FLAG (1:1000, Cell Signaling Technology, #14793S). HRP-conjugated goat anti-mouse IgG (ZB-2305; ZSGB-BIO) and goat anti-rabbit IgG (ZB-2301; ZSGB-BIO) secondary antibodies (1:10,000) were incubated for an hour at room temperature. Protein signal was detected with the Immobilon Western Chemiluminescent HRP Substrate Kit (Millipore) using ChemiDoc imaging system (Bio-Rad).

### Generation of the Gltp-knockout mice.

Targeted disruption in exons 2–4 of the *Gltp* gene on a C57BL/6JGpt background was carried out by using the CRISPR/Cas9 method by the Gempharmatech, Co. Ltd. All mice were housed in vented cages under 12-hour light-dark cycle at 23°C in a pathogen-free barrier facility. All animal experiments were conducted following the animal experimental guidelines set by the NIH Guide for the Care and Use of Laboratory Animals. For genotyping, genomic DNA was isolated from mouse tail samples using Mouse Genotyping Kit (YK-MG-100, Ubigene Biosciences). PCR amplification was performed using either the WT allele-specific primer pair (forward: 5′-CAAGAGGTTCTGCTTTGTAGCCC-3′; reverse: 5′-GGGTCTGTTATGATCCATCGTGA-3′) or the knockout allele-specific primer pair (forward: 5′-ATTGTCATGTGGCGAAGCCTC-3′; reverse: 5′-GGCCTGTATCCTACACCCTTCAA-3′).

### Immunofluorescence analysis.

Skin biopsy samples from patients and site-matched healthy controls were fixed in 4% paraformaldehyde (PFA), embedded in paraffin, and sectioned into 3-μm thick sections. The section slides were heated at 60°C for 2 hours, dewaxed in xylene, and then rehydrated in a graded ethanol series. Antigen retrieval was performed by heating the sections in 0.01M citrate buffer (pH = 6.0) for 20 minutes. The sections were permeabilized in 0.3% Triton X-100, blocked with 10% normal goat serum for 30 minutes at 37°C and incubated with diluted primary antibodies overnight at 4°C. Primary antibodies and dilutions were as follows: anti-GLTP (1:500, Novus Biologicals, NBP2-31642), anti-Ki67 (1:1000, Cell Signaling Technology, # 9449S), anti-Filaggrin (1:200, Abcam, ab218395), anti-Loricrin (1:200, Abcam, ab198994), anti-LC3B (1:1000, Abcam, ab192890), anti-SQSTM1/p62 (1:100, ZENBIO, R25788). After washing 3 times in phosphate-buffered saline (PBS), the slides were probed with goat anti-mouse or goat anti-rabbit IgG fluorescein secondary antibodies (1:100; ZSGB Biotech), coverslipped with a mounting medium containing 4’6-diamidino-2-phenylindole (DAPI, ab104139, Abcam) and visualized under a laser confocal microscope (Nikon, A1R-HD25).

For immunofluorescence analysis of cryosections and cultured cells, skin samples embedded in OCT matrix were sectioned at 5-μm thickness using a Leica cryostat (Leica, CM1950) while cells were seeded on LabTek chamber slides (Thermo Fisher Scientific). All samples were postfixed with 4% PFA, permeabilized with 0.1% Triton X-100, and blocked with normal goat serum. Then the sections were incubated with primary antibodies (anti-GLTP, 1:500, Novus Biologicals, NBP2-31642; anti-KRT14, 1:200, Sigma-Aldrich, MAB3232; KRT10, 1:2000, Abcam, ab76318; anti-ceramide, 1:100, Enzo Life Sciences, MID15B4; anti-glucosylceramide, 1:200, Glycobiotech GmbH, RAS_0011; anti-LAMP1, 1:200, Cell Signaling Technology, # 9091) overnight at 4°C, followed by secondary antibody incubation and DAPI counterstaining as described above.

### Nile red staining.

The 5-μm thickness skin cryosections were fixed in 4% PFA for 20 minutes, washed with PBS, and then stained with 1 μg/mL Nile Red solution (N3013, Sigma–Aldrich, USA) for 30 minutes at room temperature in the dark. After washing with PBS, the slides were mounted with DAPI-containing mounting medium for microscopic analysis.

### EdU incorporation assay.

The EdU (5′-ethynyl-2’-deoxyuridine) incorporation assay was conducted using the BeyoClick EdU Cell Proliferation Kit with Alexa Fluor 488 (C0071S, Beyotime) following the manufacturer’s instructions. In brief, cells were seeded in 12-well plates and exposed to EdU reagent for 2 hours at 37°C. Afterward, cells were fixed with 4% PFA and permeabilized with 0.3% Triton X-100, followed by incubation with the Alexa Fluor 488-conjugated click reaction solution for 30 minutes at room temperature. Hoechst 33342 was used to counterstain the nuclei for 15 minutes, and the cells were subsequently visualized and captured under a fluorescence microscope.

### Cell cycle analysis.

Cell cycle analysis was performed using a Cell Cycle Staining Kit (CCS012, Multi Sciences Biotech). Single-cell suspensions (5 × 10^5^) were incubated with 1 mL of propidium iodide and 10 μL of 0.1% Triton X-100 for 30 minutes at room temperature in the dark. The samples were analyzed by flow cytometry, and data were processed using FlowJo software. The fractions of cells in the G0/G1, G2, and S phases of the cell cycle were analyzed, and proliferation index (PI) was calculated (PI = (S + G2/M) / (G0/G1 + S + G2/M) × 100%).

### Transmission electron microscopy.

Skin biopsies were dissected into 1 mm³ pieces and fixed in 3% glutaraldehyde solution for 30 minutes at room temperature, followed by overnight fixation at 4°C. After 3 PBS washes, samples were post-fixed in 1% osmium tetroxide, then dehydrated through graded ethanol solution series and 100% propylene oxide. The tissues were embedded in pure EMBed 812 (90529-77-4, SPI) followed by resin, then polymerized at 60°C for 48 hours. Ultrathin sections with a thickness of 60–70 nm were cut using a Leica UC7 ultramicrotome. The sections were double stained with uranyl acetate and lead citrate, and imaged using a transmission electron microscope (HT7800, HITACHI).

### Skin permeability assay and TEWL measurements.

Skin permeability was assessed using toluidine blue dye diffusion. Euthanized newborn pups were incubated with methanol for 5 minutes, followed by washing in PBS. They were then stained with 0.1% (wt/vol) toluidine blue (G3668, Solarbio) in PBS for 2 hours, washed with PBS, and photographed to evaluate the extent of dye penetration.

TEWL was measured on the back skin of anesthetized newborn pups using the GPSkin Barrier device (G-POWER).

### RNA-seq analysis.

Total RNA of keratinocytes was isolated using Fast RNA Extraction Kit (400-100, Goonie) according to the manufacturer’s instructions. The samples were sent to Azenta Life Sciences for polyA selection, library preparation, sequencing, and data analysis. Differential expression analysis was performed using the DESeq2 Bioconductor package. Significant differential transcript expression was defined by the criteria |Log2 FC| > 1 and an adjusted *P* value < 0.05. Gene Ontology (GO) terms associated with enriched genes were identified using GOSeq (v1.34.1), with a significance threshold of *P*adj ≤ 0.05. Each treatment included 3 biological replicates. The data visualization was also using the OmicStudio tools (https://www.omicstudio.cn/tool) and the Hiplot website (https://hiplot.cn/).

### Autophagic flux.

Autophagic flux was assessed using tandem AdPlus-mCherry-GFP-LC3B (C3012, Beyotime). Primary keratinocytes were plated on LabTek chamber slides and infected with mCherry-GFP-LC3B adenovirus at a multiplicity of infection of 40 for 24 hours. Subsequently, the transfected cells were transfected with siRNA, cultured for another 48 hours in medium containing 1.3 mM CaCl_2_, and then imaged using a confocal microscope. Autophagic flux was quantified by measuring the intensity of yellow and red puncta using ImageJ software with JACoP plug-in.

### C6-NBD-GlcCer and LysoTracker labelling.

Keratinocytes on LabTek chamber slides were washed with cold Hanks’ balanced salt solution (HBSS; Gibco). A 4 μM of C6-NBD-GlcCer (HY-W356117, MCE) solution was prepared in cold HBSS by vortexing to form micelles, and cells were incubated with this solution at 4°C for 30 minutes to label the plasma membrane. Unbound probe was removed by rinsing with warm HBSS (37°C). Subsequently, HBSS was replaced with normal keratinocyte culture medium, and cells were transferred in a humidified incubator (37°C, 5% CO_2_) for 24-hour dark incubation to enable cellular uptake and trafficking of C6-NBD-GlcCer. Afterwards, LysoTracker Red DND-99 (100 nM; Yeasen) was added for incubation at 37°C for 15 minutes. Cell nuclei were then counterstained with Hoechst 33342 at 37°C for 10 minutes, and then washed, imaged using live-cell confocal microscopy immediately.

### Chemical reagents and treatments.

For autophagy inhibition, keratinocytes were treated with E64d (10 μg/mL; HY-100229, MCE) and pepstatin A (10 μg/mL; HY-P0018, MCE) for 2 hours. For GlcCer synthase inhibition, different concentrations of Eliglustat (HY-14885A, MCE) were maintained throughout 3-day differentiation period of keratinocytes induced by 1.3 mM CaCl_2_.

### Lipidomics.

Epidermal lipids from neonatal *Gltp^+/+^* and *Gltp^–/–^* mice were analyzed by liquid chromatography-tandem mass spectrometry (LC-MS/MS). Neonatal mouse epidermis was routinely isolated via overnight incubation in 0.25% dispase II at 4°C. For non-targeted lipidomics analysis, lipids were extracted using methyl tert-butyl ether (MTBE) and separated on a CSH C18 column coupled to a UPLC-Orbitrap system. Chromatography employed a gradient of acetonitrile/water (Solvent A) and acetonitrile/isopropanol (Solvent B), both containing 0.1% formic acid and 0.1 mM ammonium formate. Mass spectrometry was performed on a Q-Exactive Plus instrument in positive/negative modes (scan range, m/z 200–1800). Data were processed with LipidSearch software (Thermo Scientific) supported by isotope-labeled standards for 14 lipid classes.

For targeted analysis, lipids were extracted from frozen epidermis using a modified Bligh–Dyer method (71, 72). Quantitative profiling was conducted by LipidALL Technologies Co., Ltd. using normal-phase HPLC coupled to a Sciex QTRAP 6500 PLUS, with multiple reaction monitoring (MRM) for species-level quantification against class-specific internal standards, including Cer d18:1/15:0-d_7_, C12:0 Cer-1-P, C8-GluCer, C8-GalCer, d_3_-LacCer d18:1/16:0, Gb3 d18:1/17:0, d17:1 Sph, d17:1 S1P (Avanti Polar Lipids).

### Statistics.

Unless stated otherwise, all experiments, including Western blot, immunofluorescence, and qRT-PCR, were performed at least 3 times using independent biological replicates. Statistical analysis was performed using Prism 8.0.1 (GraphPad Software). Unless stated otherwise, data are shown as mean ± SEM, and statistical significance was evaluated by 2-tailed Student’s *t* test and Mann-Whitney test. Where applicable, multiple comparisons were corrected by controlling the False Discovery Rate with a threshold at 1% using the Benjamini, Krieger, and Yekutieli method.

### Study approval.

Human research was approved by the Clinical Research Ethics Committee of Dermatology Hospital, Southern Medical University (Guangzhou, China) (Institutional Review Board no. 2022035), and was conducted in conformity with the principles of the Declaration of Helsinki. Written informed consent from the patients or their guardians was obtained for both sample collection and publication of clinical photographs, and the records of such consent have been duly retained by the authors. All animal studies were conducted according to standard animal guidelines and approved by the animal welfare committee of South China Agricultural University (Guangzhou, China) (approval number 2025D003).

### Data availability.

Supporting data relevant to the main manuscript and supplement are available in the Supporting Data Values file. The raw WES data, bulk RNA-seq data as well as the lipidomic data reported in this publication have been deposited in the National Genomics Data Center under BioProject accession number PRJCA048709 (https://ngdc.cncb.ac.cn/gsa-human).

## Author contributions

ZZ, SH, SZ, and SP designed and conducted experiments. AJ, EAJ, SB, CY, and ZL enrolled patients and collected clinical samples. ZZ and SH analyzed the data and generated figures. KL conducted the bioinformatic analysis. ZZ and HW wrote the manuscript. HW and ZL guided the research, provided financial support, and supervised the study. The order of the co–first and co–corresponding authors names was determined by the extent of contribution to the study. All authors read and approved the final version of the manuscript.

## Conflict of interest

The authors have declared that no conflict of interest exists.

## Funding support

National Natural Science Foundation of China (grant 82373500 to HW, grant 82373459 to ZL).

- European Union’s Horizon 2020 research and innovation program under the SolveRD project (grant 779257 to AJ and SB).
- National Institute for Health and Care Research (NIHR) Manchester Biomedical Centre (grant NIHR203308).

## Supplementary Material

Supplemental data

Unedited blot and gel images

Supporting data values

## Version 1 02/05/2026

In-Press Preview

## Version 2 04/15/2026

Electronic publication
