# Supplementary material for: Biallelic GLTP mutations cause nonsyndromic epidermal differentiation disorder via disrupted epidermal glucosylceramide transport
Source: J Clin Invest. 2026 Feb 5;136(8):e198835. doi: 10.1172/JCI198835 (PMC13078884; doi:10.1172/JCI198835)
Supplement: Supplemental data [file jci-136-198835-s153.pdf]

SUPPLEMENTARY FIGURES AND TABLES

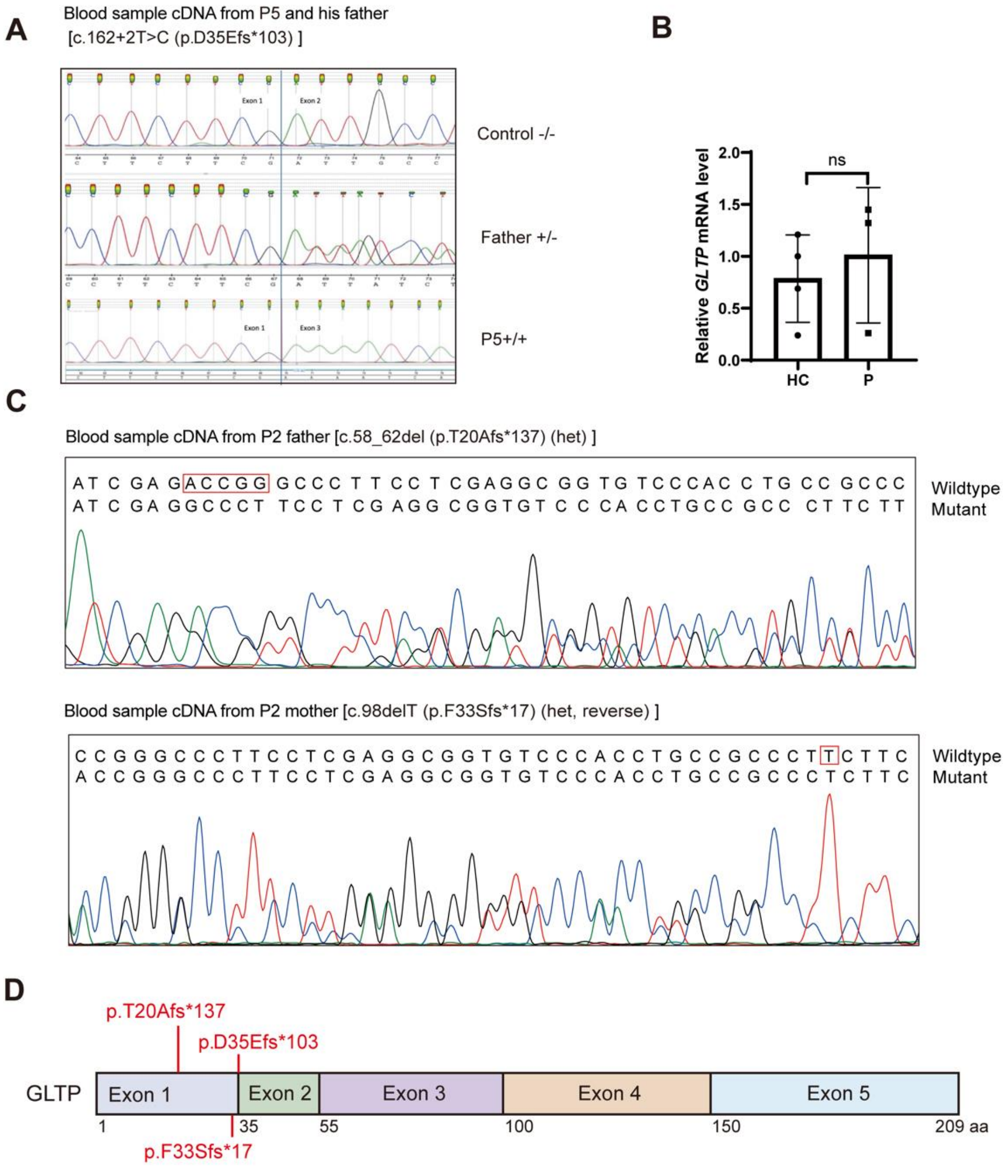

Supplemental Figure 1. Transcriptional analysis and localization of the GLTP variants. (A) Reverse transcription-PCR of RNA isolated from blood sample of the P5 and his father followed by sequencing of the GLTP transcript. (**B**) RT-qPCR showed the frameshift variants in GLTP in keratinocytes do not result in nonsense-mediated mRNA decay compared to the normal control. ns, not significant (Student's t test). (**C**) Reverse transcription-PCR of RNA isolated from blood sample of the parents from P2 followed by sequencing of the GLTP transcript. (**D**) Schematic representation of exons of human GLTP protein.

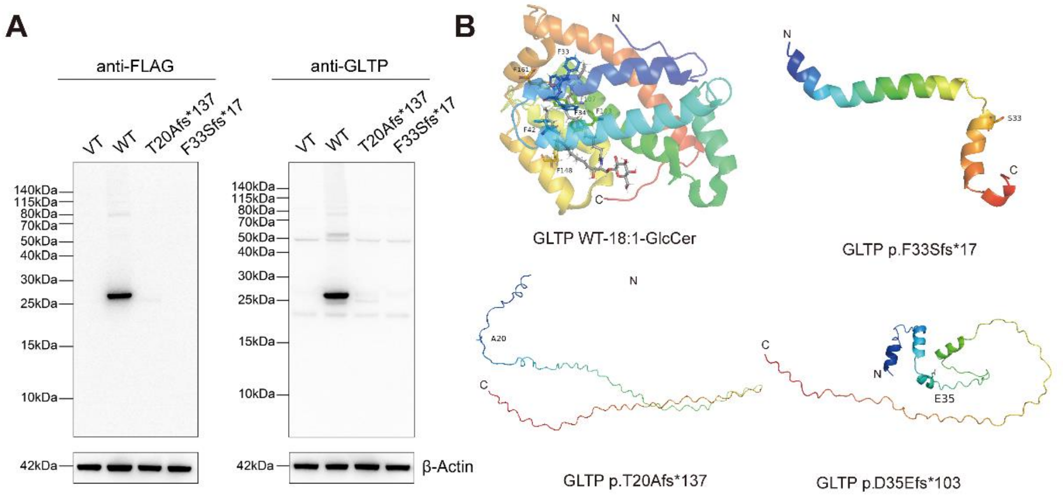

Supplemental Figure 2. Frameshift mutations lead to unstable GLTP protein and disruption of the glycolipid-binding pocket. (**A**) Western blot of the protein extracts from 293T cells transfected with NH2-terminally FLAG-tagged GLTP constructs (wild-type, WT; or frameshift mutants, T20Afs137/F33Sfs17) or empty vector (VT). Lysates were immunoblotted with anti-FLAG (left) or anti-GLTP (right) antibody. β-Actin served as a loading control. (**B**) Crystal structure of human GLTP (cartoon model with important residues highlighted as sticks) in complex with N-oleoyl-containing glucosylceramide (18:1 GlcCer) (stick model) (PDB:3S0K). The hydrophobic pocket of GLTP, which encapsulates the lipid acyl chain, is comprised of seven phenylalanine residues (F33, F34, F42, F103, F107, F148, and F161), highlighted as sticks. Glucosylceramide atoms are shown in sticks and colored gray, red and blue for carbon, oxygen and nitrogen, respectively. AlphaFold-predicted structures for the GLTP mutant proteins p.F33Sfs*17, p.T20Afs*137 and p.D35Efs*103.

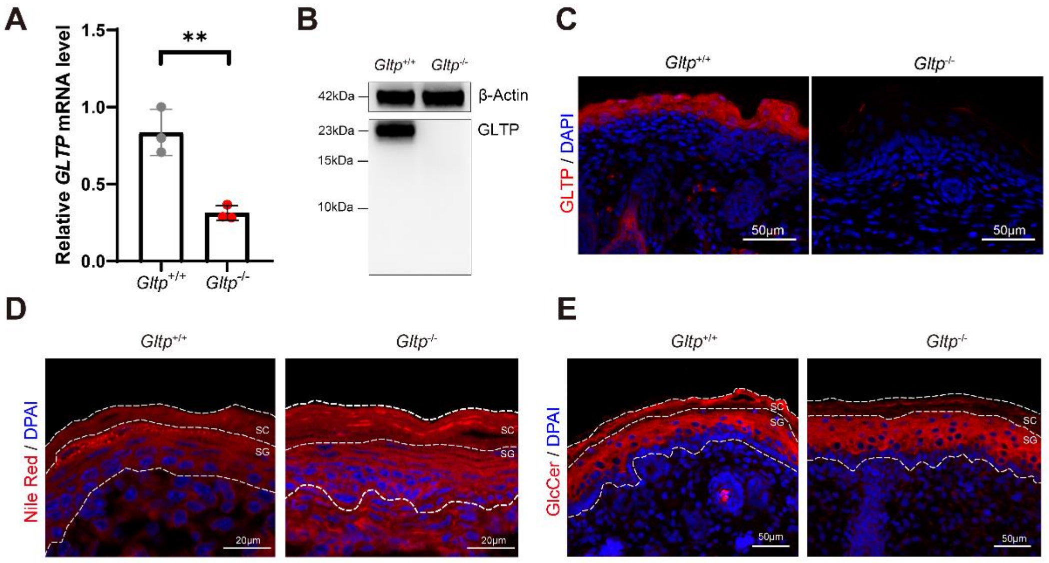

Supplemental Figure 3. Validation of GLTP knockout mice and examination of the epidermal lipid organization. (**A**) RT-qPCR of GLTP expression in the epidermis from newborn *Gltp*$^{+/+}$ and *Gltp*$^{-/-}$ mice. n = 3, ***P* < 0.01 (Student's t test). (**B**) Western blot showed lack of GLTP protein expression in the epidermis from *Gltp*$^{+/+}$ and *Gltp*$^{-/-}$ mice newborns. (**C**) Immunohistochemical staining for GLTP in the dorsal skin of newborn mice. (**D**) Fluorescent staining of neutral lipids with Nile Red. (**E**) Immunohistochemical staining for glucosylceramide (GlcCer). SC, stratum corneum; SG, stratum granulosum.

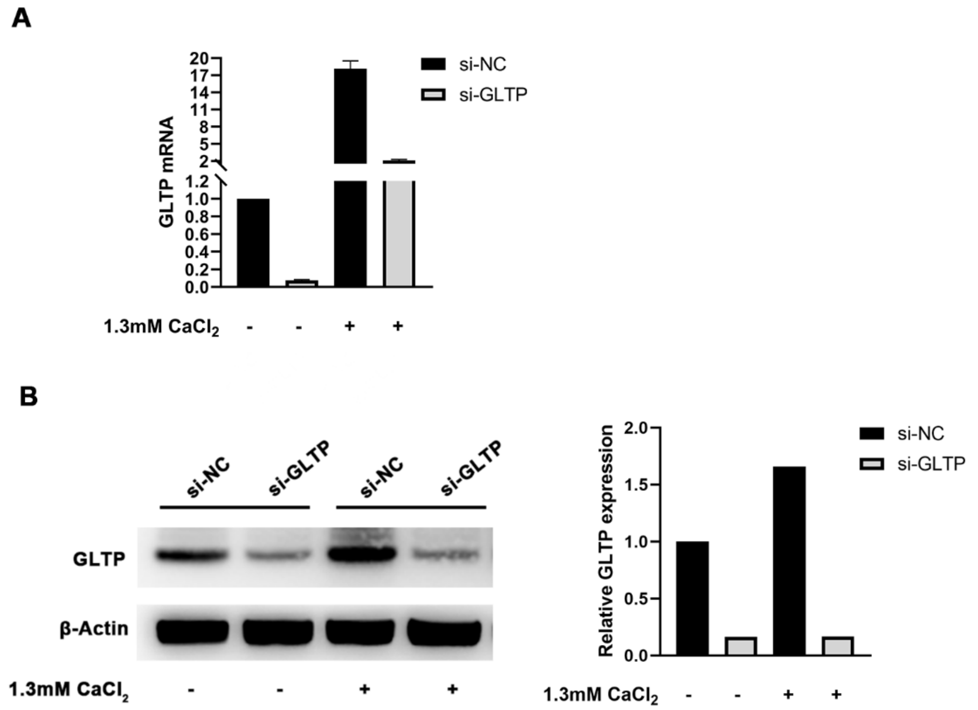

Supplemental Figure 4. Validation of GLTP knockdown efficiency. (**A**) RT-qPCR analysis of GLTP mRNA expression in keratinocytes under undifferentiated and differentiated conditions with si-NC or si-GLTP. (**B**) Left, Western blot of GLTP in undifferentiated and differentiated keratinocytes transfected with si-NC or si-GLTP. β-Actin is shown as a loading control. Right, Quantification of Western blot analysis for GLTP.

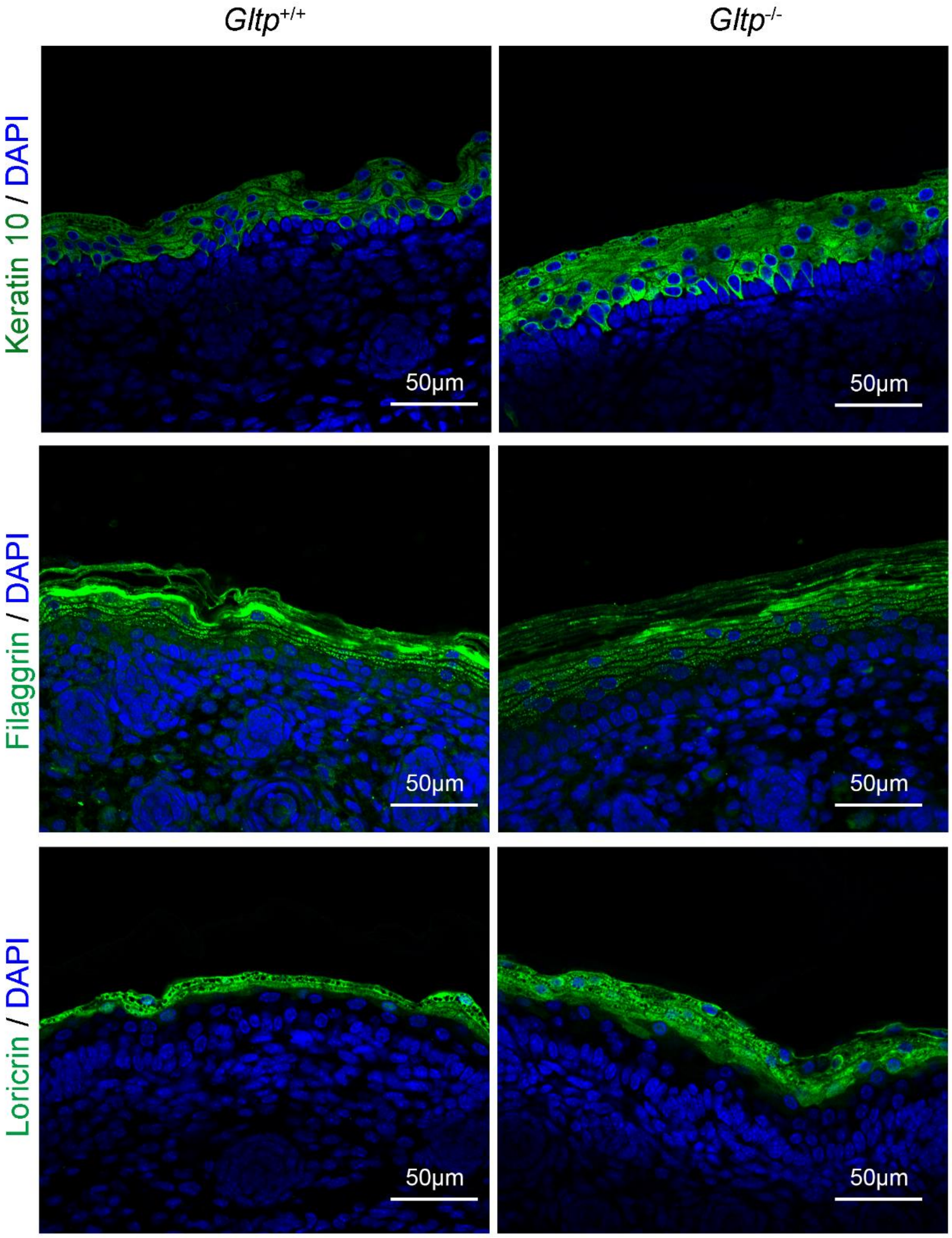

Supplemental Figure 5. Immunohistochemical staining of keratin10, filaggrin, and loricrin in skin sections from the epidermis from *Gltp*$^{+/+}$ and *Gltp*$^{-/-}$ mice newborns. SC, stratum corneum; SG, stratum granulosum.

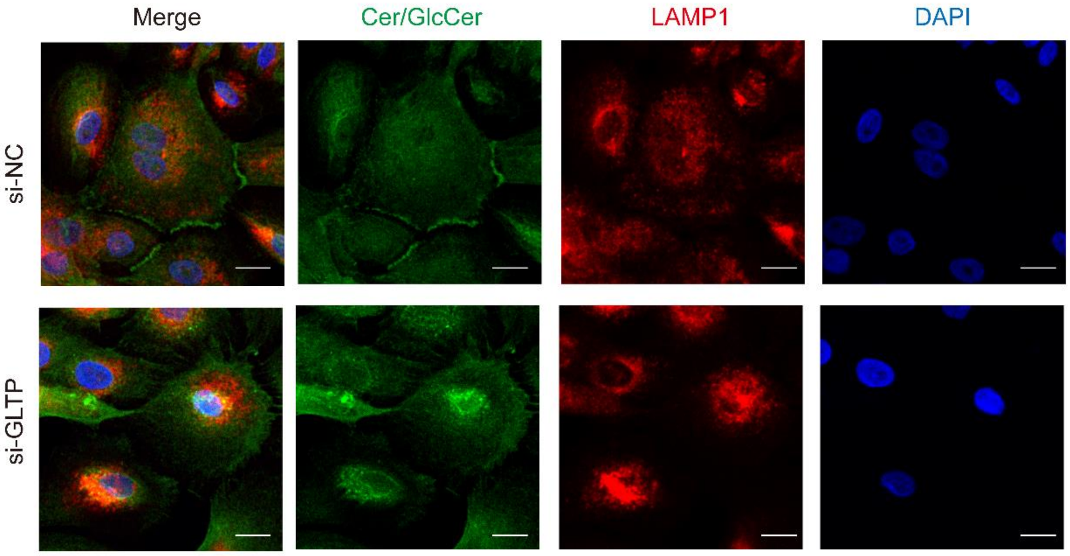

Supplemental Figure 6. Double immunofluorescence for ceramide and LAMP1 in differentiated human epidermal keratinocytes. Scale bars: 10 μm.

Supplemental Table 1. Primers used for Sanger sequencing.

Target	Primer sequence (5'→3')
GLTP-exon 1	F: CGAAAGAGAACCGTGACCAAC<br>R: TGACATGTTTAGAGCGGAGAGG

Supplemental Table 2. Primers used for quantitative real-time PCR.

Target	Primer sequence (5'→3')
GAPDH	F: AGGTCGGAGTCAACGGATTT<br>R: ATGAAGGGGTCATTGATGGCA
GLTP	F: CTGCTGGCCGAACACTTG<br>R: TTTTCGTGATGTTGCCGCTT
K10	F: TCCCAACTGGCCTTGAAACAA<br>R: GCAACTGTTCTTCCAGAGCG
IVL	F: TCCTCCAGTCAATACCCATCAG<br>R: CAGCAGTCATGTGCTTTTCCT
FLG	F: CGGCAAATCCTGAAGAATCCA<br>R: GTGCTTTCTGTGCTTGTGTCC
LOR	F: GCTTTGGGCTCTCCTTCCTT<br>R: AGGTCTTCACGCAGTCCAC
GLTP-exon 1 (mouse)	F: ACCTGCTCAAGCCGCTG<br>R: CAGGTGAGCCACAGCCTC
