# Supplementary figures and images for: Biallelic GLTP mutations cause nonsyndromic epidermal differentiation disorder via disrupted epidermal glucosylceramide transport
Source: J Clin Invest. 2026 Feb 5;136(8):e198835. doi: 10.1172/JCI198835 (PMC13078884; doi:10.1172/JCI198835)

Figure 2B

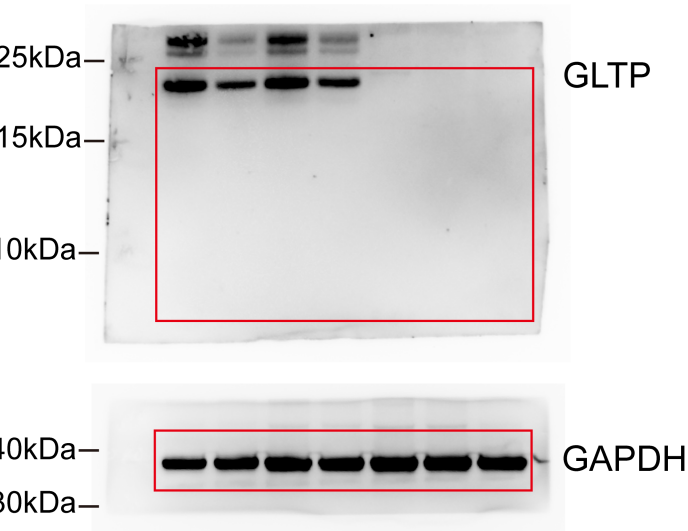

Figure 9B

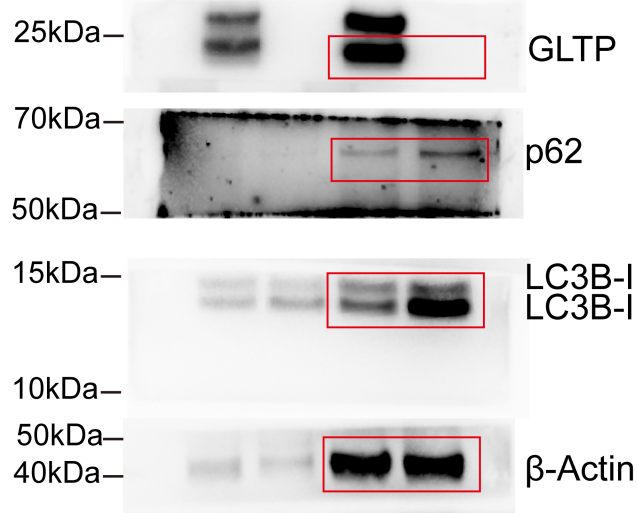

Figure 9C

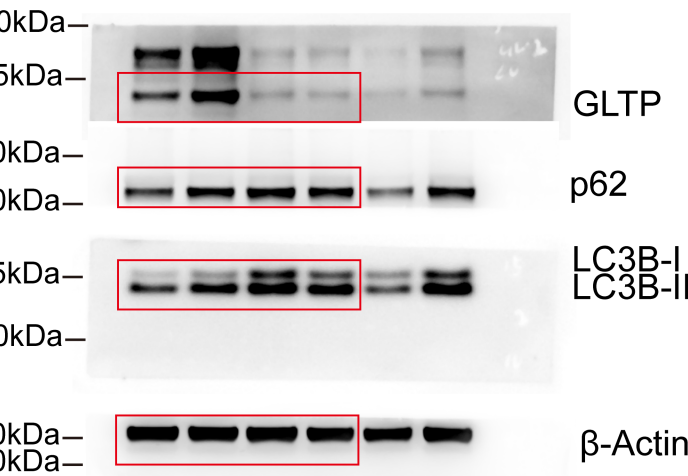

Figure 9G

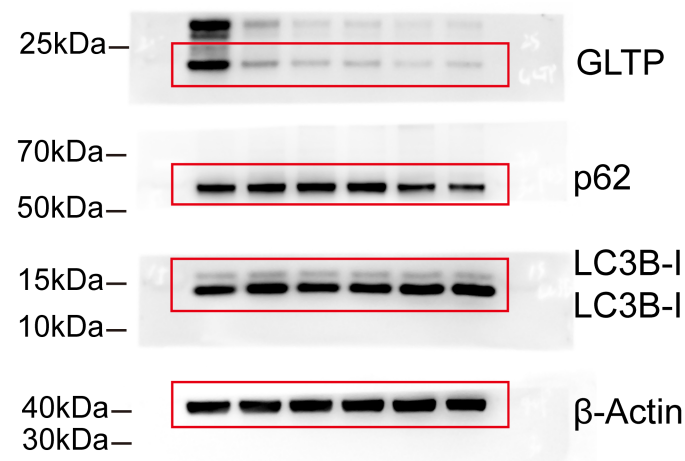

Figure S3B

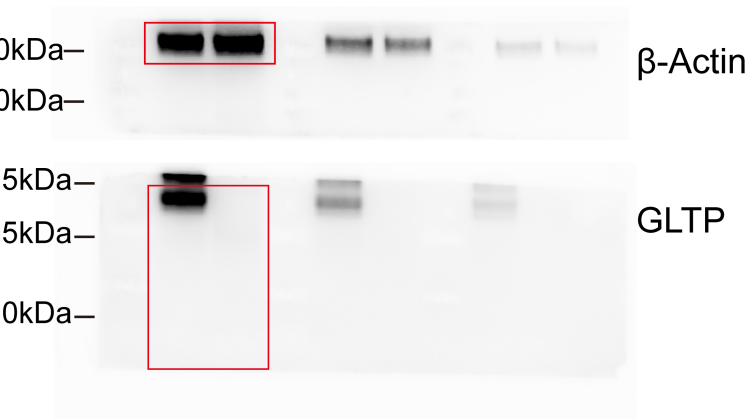

Figure S4B

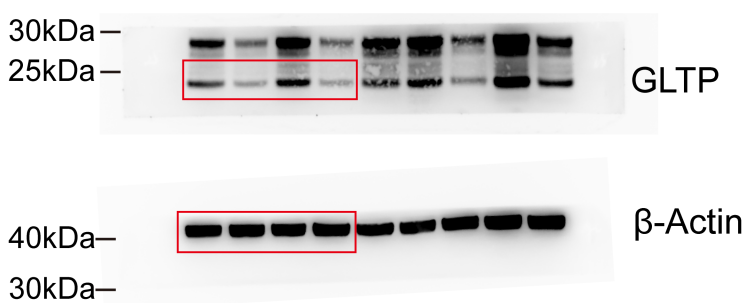

Supplement: Unedited blot and gel images [file jci-136-198835-s154.pdf]
